# Supplementary material for: Exploring the relation between modelled and perceived workload of nurses and related job demands, job resources and personal resources; a longitudinal study
Source: PLoS One. 2021 Feb 26;16(2):e0246658. doi: 10.1371/journal.pone.0246658 (PMC7909623; doi:10.1371/journal.pone.0246658)
Supplement: Attachment — Submitted filename: moddelled.pdf_commented.pdf [file pone.0246658.s002.pdf]

PLOS ONE

Exploring the relation between modelled workload and perceived workload of nurses and related job demands, job resources and personal resources, a longitudinal study

--Manuscript Draft--

**Manuscript Number:**	PONE-D-20-08785
**Article Type:**	Research Article
**Full Title:**	Exploring the relation between modelled workload and perceived workload of nurses and related job demands, job resources and personal resources, a longitudinal study
**Short Title:**	Exploring the relation between modelled workload and perceived workload of nurses, a longitudinal study
**Corresponding Author:**	W.F.J.M. van den Oetelaar, MSc<br>Universitair Medisch Centrum Utrecht<br>Utrecht, NETHERLANDS
**Keywords:**	nurse workload, nurse staffing, job demands, workload management, nursing intensity
**Abstract:**	Aim<br><br>Calculating an objective, modelled workload, exploring the relation between this modelled workload and workload as perceived by nurses and studying the effects of certain job demands, job resources and personal resources.<br><br>Design<br><br>Academic hospital in the Netherlands. Six surgical wards, capacity 15-30 beds. Data collected over 15 consecutive day shifts.<br><br>Methods<br><br>Modelled workload is calculated as a ratio of required care time, based on patient characteristics, baseline care time and time for non-patient related activities, and allocated care time, based on the amount of available nurses. Both required and allocated care time are corrected for nurse proficiency. Five dimensions of perceived workload were determined by questionnaires. Both the modelled and the perceived workloads were measured on a daily basis. Linear mixed effects models study the longitudinal relation between this modelled and workload as perceived by nurses and the effects of personal resources, job resources and job demands. ANOVA and post-hoc tests were used to identify differences in modelled workload between wards.<br><br>Results<br><br>Modelled workload varies roughly between 70 and 170%. Significant differences in modelled workload between wards were found but confidence intervals were wide. Modelled workload is positively associated with all five perceived workload measures (work pace, amount of work, mental load, emotional load, physical load). In addition to modelled workload, the job resource support of colleagues and job demands time spent on direct patient care and time spent on registration had the biggest significant effects on perceived workload.<br><br>Conclusions<br><br>The modelled workload can be used to detect differences in workload between wards, which may be useful in distributing workload more evenly in order prevent issues in the domain of e.g. organizational justice. Average patient characteristic profiles per admission day can be composed for treatments such as operations to predict an indication for workload. Extra effort in team building is likely to have a positive effect on perceived workload. A good balance between time spent on direct patient care and registration may also benefit perceptions of workload. The findings of this research can help nursing management in allocating resources and directing their attention to the most relevant factors for balancing workload.

Powered by Editorial Manager® and ProduXion Manager® from Aries Systems Corporation

Order of Authors:	Miranda van den Oetelaar, MSc
	Corne Roelen
	Wilko Grolman
	Rebecca Stellato
	Willem Van Rhenen
**Additional Information:**	
**Question**	**Response**
**Financial Disclosure**<br><br>Enter a financial disclosure statement that describes the sources of funding for the work included in this submission. Review the submission guidelines for detailed requirements. View published research articles from *PLOS ONE* for specific examples.<br><br>This statement is required for submission and **will appear in the published article** if the submission is accepted. Please make sure it is accurate.<br><br>**Unfunded studies**<br>Enter: *The author(s) received no specific funding for this work.*<br><br>**Funded studies**<br>Enter a statement with the following details:<br>• Initials of the authors who received each award<br>• Grant numbers awarded to each author<br>• The full name of each funder<br>• URL of each funder website<br>• Did the sponsors or funders play any role in the study design, data collection and analysis, decision to publish, or preparation of the manuscript?<br>• **NO** - Include this sentence at the end of your statement: *The funders had no role in study design, data collection and analysis, decision to publish, or preparation of the manuscript.*<br>• **YES** - Specify the role(s) played.<br><br>* typeset	NO. The funders had no role in study design, data collection and analysis, decision to publish, or preparation of the manuscript.
**Competing Interests**<br><br>Use the instructions below to enter a competing interest statement for this submission. On behalf of all authors,	The authors have declared that no competing interests exist.

disclose any competing interests that could be perceived to bias this work—acknowledging all financial support and any other relevant financial or non-financial competing interests.<br><br>This statement **will appear in the published article** if the submission is accepted. Please make sure it is accurate. View published research articles from *PLOS ONE* for specific examples.<br><br>**NO authors have competing interests**<br><br>Enter: *The authors have declared that no competing interests exist.*<br><br>**Authors with competing interests**<br><br>Enter competing interest details beginning with this statement:<br><br>*I have read the journal's policy and the authors of this manuscript have the following competing interests: [insert competing interests here]*<br><br>* typeset	
**Ethics Statement**<br><br>Enter an ethics statement for this submission. This statement is required if the study involved:<br><br>• Human participants<br>• Human specimens or tissue<br>• Vertebrate animals or cephalopods<br>• Vertebrate embryos or tissues<br>• Field research<br><br>Write "N/A" if the submission does not require an ethics statement.<br><br>General guidance is provided below. Consult the submission guidelines for detailed instructions. **Make sure that all information entered here is included in the Methods section of the manuscript.**	The study guaranteed the privacy of involved staff and patients. The study protocol was reviewed and approved by the medical ethical review board of the UMC Utrecht, protocol number 14-165/C.

**Format for specific study types**<br><br>**Human Subject Research (involving human participants and/or tissue)**<br>• Give the name of the institutional review board or ethics committee that approved the study<br>• Include the approval number and/or a statement indicating approval of this research<br>• Indicate the form of consent obtained (written/oral) or the reason that consent was not obtained (e.g. the data were analyzed anonymously)<br><br>**Animal Research (involving vertebrate animals, embryos or tissues)**<br>• Provide the name of the Institutional Animal Care and Use Committee (IACUC) or other relevant ethics board that reviewed the study protocol, and indicate whether they approved this research or granted a formal waiver of ethical approval<br>• Include an approval number if one was obtained<br>• If the study involved *non-human primates*, add *additional details* about animal welfare and steps taken to ameliorate suffering<br>• If anesthesia, euthanasia, or any kind of animal sacrifice is part of the study, include briefly which substances and/or methods were applied<br><br>**Field Research**<br><br>Include the following details if this study involves the collection of plant, animal, or other materials from a natural setting:<br>• Field permit number<br>• Name of the institution or relevant body that granted permission	
**Data Availability**<br><br>Authors are required to make all data underlying the findings described fully available, without restriction, and from the time of publication. PLOS allows rare exceptions to address legal and ethical concerns. See the PLOS Data Policy and FAQ for detailed information.	Yes - all data are fully available without restriction

Powered by Editorial Manager® and ProduXion Manager® from Aries Systems Corporation

A Data Availability Statement describing where the data can be found is required at submission. Your answers to this question constitute the Data Availability Statement and **will be published in the article**, if accepted.<br><br>**Important:** Stating 'data available on request from the author' is not sufficient. If your data are only available upon request, select 'No' for the first question and explain your exceptional situation in the text box.<br><br>Do the authors confirm that all data underlying the findings described in their manuscript are fully available without restriction?	
**Describe where the data may be found in full sentences. If you are copying our sample text, replace any instances of XXX with the appropriate details.**<br><br>• If the data are **held or will be held in a public repository**, include URLs, accession numbers or DOIs. If this information will only be available after acceptance, indicate this by ticking the box below. For example: *All XXX files are available from the XXX database (accession number(s) XXX, XXX.)*.<br>• If the data are all contained **within the manuscript and/or Supporting Information files**, enter the following: *All relevant data are within the manuscript and its Supporting Information files.*<br>• If neither of these applies but you are able to provide **details of access elsewhere**, with or without limitations, please do so. For example:<br><br>*Data cannot be shared publicly because of [XXX]. Data are available from the XXX Institutional Data Access / Ethics Committee (contact via XXX) for researchers who meet the criteria for access to confidential data.*<br><br>*The data underlying the results presented in the study are available*	All relevant data are within the manuscript and its Supporting Information files.If more information is required, it is available upon request, with the corresponding author

from (include the name of the third party and contact information or URL).<br>• This text is appropriate if the data are owned by a third party and authors do not have permission to share the data.<br><br>* typeset	
Additional data availability information:	

Manuscript Revised
Click here to access/download;Manuscript;Manuscript 5 relation obj subj job dem 20200330 Revised.docx

Title page

Title

Exploring the relation between modelled workload and perceived workload of nurses and related job demands, job resources and personal resources, a longitudinal study

Authors

W.F.J.M. van den Oetelaar [1*]

C.A.M. Roelen[4]

W. Grolman[5]

R.K. Stellato[2]

W. van Rhenen[3,4]

*Corresponding author:

Email: W.f.j.m.vandenoetelaar@umcutrecht.nl

[1]Division of Internal Medicine, University Medical Center Utrecht, University of Utrecht , Utrecht, the Netherlands

[2]Julius Center for Health Sciences and Primary Care, University Medical Center Utrecht, University of Utrecht , Utrecht, the Netherlands

[3]Center for Human Resource Organization and Management Effectiveness, Business University Nyenrode, Breukelen, the Netherlands

[4]ArboUnie Occupational Health Service, Utrecht, the Netherlands

[5]Division of Surgical Specialties, University Medical Center Utrecht, University of Utrecht , Utrecht, the Netherlands

WO and WG conceived the study. WO, WG, WR, CR and RS participated in its design. WO gathered and cleaned data, coordinated the study and drafted this manuscript. RS performed statistical analysis. CR helped to draft the manuscript. All authors revised the manuscript drafts and approved the final manuscript.

Competing interests

All authors declare: no support from any organization for the submitted work; no financial relationships with any organizations that might have an interest in the submitted work in the previous three years; no other relationships or activities that could appear to have influenced the submitted work.

Abstract

Aim

Calculating an objective, modelled workload, exploring the relation between this modelled workload and workload as perceived by nurses and studying the effects of certain job demands, job resources and personal resources.

Design

Academic hospital in the Netherlands. Six surgical wards, capacity 15-30 beds. Data collected over 15 consecutive day shifts.

Methods

Modelled workload is calculated as a ratio of required care time, based on patient characteristics, baseline care time and time for non-patient related activities, and allocated care time, based on the amount of available nurses. Both required and allocated care time are corrected for nurse proficiency. Five dimensions of perceived workload were determined by questionnaires. Both the modelled and the perceived workloads were measured on a daily basis. Linear mixed effects models study the longitudinal relation between this modelled and workload as perceived by nurses and the effects of personal resources, job resources and job demands. ANOVA and post-hoc tests were used to identify differences in modelled workload between wards.

Results

Modelled workload varies roughly between 70 and 170%. Significant differences in modelled workload between wards were found but confidence intervals were wide. Modelled workload is positively associated with all five perceived workload measures (work pace, amount of work, mental load, emotional load, physical load). In addition to modelled workload, the job resource support of colleagues and job demands time spent on direct patient care and time spent on registration had the biggest significant effects on perceived workload.

Conclusions

The modelled workload can be used to detect differences in workload between wards, which may be useful in distributing workload more evenly in order prevent issues in the domain of e.g. organizational justice. Average patient characteristic profiles per admission day can be composed

for treatments such as operations to predict an indication for workload. Extra effort in team building is likely to have a positive effect on perceived workload. A good balance between time spent on direct patient care and registration may also benefit perceptions of workload. The findings of this research can help nursing management in allocating resources and directing their attention to the most relevant factors for balancing workload.

Introduction

In healthcare, there is an increasing pressure on maintaining a high quality of care whilst containing expenditure [1]. Healthcare expenditure is increasing due to factors such as technological progress and an aging population with more chronic conditions [2, 3]. Healthcare providers are expected to maintain a high quality of care under increasing demand, with the same or less funding [4-6]. There is a risk that under these circumstances, nurses' workload will increase to alarming levels. Workload is considered unacceptable, if nurses are not able to meet patients' needs, physical as well as emotional [7] or if nurses' health is at risk. There is overwhelming evidence of the adverse effects of high workload of nurses. There is a direct relation between nurses' workload and patient satisfaction [8], patient outcomes [9-14] and nurse reported quality of care and performance [15-17]. High workload is also a predictor for nurses' job dissatisfaction, stress, burnout [18-20] and absenteeism [21], and has generally been shown to have a negative effect on job outcomes [22] and nurses' intention to leave [23, 24]. High turnover of nursing staff results in higher costs for training of new nurses, or hiring of temporary staff [11, 25]. Nurses also report that when workload is high, they cannot deliver all care that they believe they should [26]. Important tasks, such as tending to patients' emotional and psychological needs, are left undone, which leaves nurses feeling unsatisfied with their job and occupation, which in turn leads to an increasing intent to leave, lower reported quality of care and deteriorating patient satisfaction [26].

Managing workload will therefore become increasingly important in preventing the vicious cycle where nurses are leaving the job due to high workload, which leads to a greater shortage and an even higher workload.

Despite the fact that a high workload can have such far-reaching practical implications, the concept of workload is not always clearly defined in research. It is often measured by using crude staffing ratios or determined by questionnaires regarding perceived workload. Also, many studies examine only one dimension of workload, such as perceived mental load or amount of work [27]. Alghamdi [28] defined workload as the amount of time and care that a nurse can devote (directly and indirectly) towards patients, workplace and professional development. This definition covers direct and indirect patient care but also non-nursing activities such as meetings and attending seminars. Alghamdi advocated a holistic approach and determined five defining attributes of nursing workload: the amount of nursing time spent on nursing care known as patient acuity; the level of nursing competency; the weight of nursing intensity (direct patient care); all the physical, mental and emotional efforts; and the ability of the nurse to change the plan (complexity of care).

Holden [29] describes three different types of perceived workload: task-level, job-level and unit-level workload. Unit-level workload considers the balance between patient acuity and staffing, job-level workload entails general and specific demands of the job such as the general amount and difficulty of the work and the amount of concentration required to do it, and task-level workload relates to the demands and resources for a specific task such as medication preparation. These workload types describe different dimensions of workload and each type of workload has a specific effect on burnout, job dissatisfaction and the likelihood of medication errors. Holden's study did not consider emotional and physical workload, but recommended also taking these into account. In the Netherlands, the Questionnaire on the Experience and Evaluation of Work (QEEW [30]), an evidence based questionnaire, is widely used by Dutch occupational health services to measure the (psychosocial) working environment. It defines four types of workload: work pressure (a combination of work pace and amount of work), emotional load, mental load and physical effort.

The well-known Job Demands and Resources (JDR) model [31] also considers workload. The JDR model describes a health impairment process where high job demands lead to exhaustion and burnout. Job demands are those aspects of a job that require effort. On the other hand, the JDR model describes a motivational process where job resources play a crucial role. Job resources are functional in achieving work goals, reduce job demands and the associated physiological and psychological costs and stimulate personal growth and development [31]. Workload is considered a job demand in the JDR model; prolonged periods of high workload can lead to burnout, especially if there are insufficient job resources to counterbalance the effects of the job demands [31-33].

A previous study by our group [34] presented a method to calculate and predict nurses' workload by means of objective measures. Subsequent studies yielded a set of patient characteristics - developed by expert nurses- that were expected to be significantly related to care time [35, 36]. Significance and care time estimates of these characteristics were determined [36] and in addition it was also specified how much time is spent on patients regardless of these characteristics [36] and how much time is spent on non-patient related activities [37]. The importance of including nurse proficiency in workload calculations was also demonstrated [36].

In the study presented in this paper, we build on the findings of this previous research. Both objective (calculated, modelled) and subjective (perceived, self-reported) workload measures are included in the study.

The aim is to understand the relation between objective, modelled workload and workload as it is perceived by nurses, and to test the effects of several relevant demands and resources on this relation.

This paper considers the effects of four job demands, three job resources and two personal resources on the relation between modelled and perceived workload.

Perceived interruptions, perceived equal work distribution, time spent on registration and time spent on direct patient care are considered job demands in the context of this study. Dutch occupational health surveys, used by occupational health and safety services for preventive medicine purposes, often include questions on work interruptions and equal work distribution. There is evidence that strongly relates the number of work interruptions to patient outcomes [38, 39] and nurses' workload [40, 41]. Equal work distribution has not been related to perceived workload in literature before. Registration required by the government, insurance companies and hospital management, is one of the reasons nurses experience a high workload. Myny [41] found a link between perceived workload and the number of mandatory registrations. Van Bogaert [7] also identifies a growing problem of additional registration. There are also indications that increased administration burden competes with the time intended to be spent on patient care; Khademi [42] stated that one of the important sources of increased nursing workload was an overemphasis of managers on frequent report writing, which competed with patient care delivery. Workload may also be experienced differently if the proportion of time that nurses can spend on direct patient care is low, due to either increased registration or other reasons.

The JDR model postulates that the health impairment process can be mitigated by job resources and personal resources[31] . These resources help to achieve goals and stimulate personal development, resulting in intrinsic motivation and engagement [31, 33, 43-46]. The present study will include three job resources: support from colleagues, support from management, and the proportion of registered nurses on the ward. Yanchus [47] found that the job resource teamwork (i.e, colleagues helping and backing each other up) counterbalances the effects of understaffing and high workload. Similarly, Sexton's research [48] found that workload pressures can be offset by a positive nursing team environment on a unit. This indicates that teamwork is an important factor in perceived workload. Van Bogaert et al. [7] found the same results in their study on the predictors of burnout, work engagement, nurse reported job outcomes and quality of care. Van Bogaert et.al. [49] also reported that hospital management directly influenced nurses' perceived workload. In MacPhee's study on the impact of heavy perceived workload of nurses on patient and nurse outcomes [14], it was noted that unit level leaders in particular could influence

perceived quality of care and job outcomes by monitoring and responding to workload demands. In addition, there is much evidence that a nursing staff mix with a large proportion of registered nurses results in better patient outcomes [39, 50, 51]. The effect of the skill mix of nurses on perceived workload has not been extensively studied, although there is evidence that skill mix is an important factor when considering workload [52] and that a lower proportion of registered nurses on a ward is associated with increased workload [53]. In the current paper, the proportion of registered nurses on the ward is included and regarded as a job resource.

Two personal resources have been included in the current study: self-efficacy and proficiency. Spence Laschinger [54] found a significant correlation between workload (as one of the areas of work life) and occupational coping self-efficacy, defined as the self-appraisal of one's capability to cope with occupational burden in the workplace [55]. When the areas of work life, such as workload, are balanced, this has a positive effect on occupational coping self-efficacy. In the hospice setting, the stress of staff shortages decreased with increasing self-efficacy [56]. Schmidt et al. [44] found that self-efficacy had a direct effect on job strain in nursing homes, but they did not find evidence for an interaction between self-efficacy and perceived workload. Brunetto [57] found that self-efficacy reduced the effects of stress and enhanced job satisfaction, and herewith reduced nurses' intentions to leave the job; however, they did not study the effects on perceived workload.

Nurses' education and experience are related to clinical expertise [58]. A well-educated, experienced nurse may be able to handle workload better than a novice nurse. This study will test whether nurses with more working experience and who are considered to be more proficient experience workload differently than other nurses.

We will test the following hypotheses:

- *Hypothesis 1*: there is a correlation between the modelled (objective) workload measure and perceived (subjective) workload
- *Hypothesis 2*: perceived workload is lower when personal resources self-efficacy and nurse proficiency are higher
- *Hypothesis 3*: the job resources support from management, support from colleagues and proportion of registered nurses moderate the relation between modelled and perceived workload
- *Hypothesis 4*: the job demands proportion of direct patient care, proportion of administration, work interruptions and perceived equality of work distribution moderate the relation between modelled and perceived workload.

Method

Study setting

The research took place in an academic hospital in the Netherlands (private hospital). Six surgical wards were included (2 wards with 15 beds, 4 wards with 30 beds). The study focused on the day shift workload, because this is the shift during which the most nurses are required and most nursing activities are performed, some of which only occur in the dayshift.
Weekends were excluded because the task mix and staffing are very different in weekends and cannot be compared to the day shifts of regular weekdays.

Participants

All registered nurses and nurse students working on the study wards were included in the study. Ward managers were excluded because they do not perform patient-related activities. The study focused on workload of nurses: other professionals such as physicians, physician-assistants, and paramedics were not included in this study.

Factors that consider employees other than nurses (such as nurse-physician relationships, support from logistic teams), factors that cannot be influenced by nurse management of the ward (such as social support at home) or factors that require major investments (such as ward layout and number of single rooms in a ward) were not included.

Design

In this research, five different measures of perceived workload are included: work pace, amount of work, mental workload, emotional workload and physical workload. The job demands (interruptions, work distribution, time for registration, time for direct patient care) and job resources (support from colleagues, support from management, the proportion of registered nurses on the ward) are included in the analyses as potential effect modifiers of the relation between modelled and perceived workload. Effects will be explored separately for each outcome measure. Error! Reference source not found. presents a visualization of the model to be studied.

Figure 1: Studying the relation between modelled and perceived workload (our hypotheses)

Data collection

Data was collected in four ways:

1. An observational work sampling study of the activities of nurses was done during fifteen consecutive day shifts. Details on the study can be found in a previous publication of the study team [37]. This study yielded estimates of time spent on direct patient care, collective patient care, general tasks and other tasks.
2. During the observational study period, a previously determined set of relevant patient characteristics were registered each dayshift for all patients admitted on the study wards [36]. This information was combined with the work sampling results and a linear mixed effects model was used to determine significance and care time estimates for each characteristic [35, 36].
3. During the observational study, each nurse on duty during the dayshift was asked to fill out a short questionnaire at the end of each dayshift. The questionnaire contained questions on job demands, job resources, engagement, quality of care and stress.
4. Three weeks prior to the observational study, all nurses employed on the study wards (as registered nurses or students) were asked to fill out an extensive questionnaire on job demands, job resources, personal resources and personal and job characteristics.

Measures

Outcome measures: perceived workload

Perceived workload was measured with items derived from the Questionnaire on the Experience and Evaluation of Work (QEEW), which is widely used by Dutch occupational health services to measure the (psychosocial) working environment [44]. The five measures for perceived workload were:

'Did you have too much work to do today', reflecting the amount of work;

'Did you have to work very fast today', reflecting work pace;

'Did you consider your work mentally very challenging today', reflecting mental workload;

'Did your work demand a lot from you emotionally today', reflecting emotional workload;

'Did you find your work physically strenuous today', reflecting physical workload.

Each item had a five-point response scale ranging from 'Not at all' (1) to 'Very often' (5). All measurements were done during the work sampling study period, on a daily basis at the end of the shift.

Independent variable: modelled workload

As mentioned, previous work of this study team contained a study design for developing a workload management method [34]. This method consisted of 7 steps. In step 1, a group of expert nurses composed a list of fifteen patient characteristics they expected to be most relevant to care time. Subsequently, an observational study on nurses' activities (step 2) measured how much time nurses spent on direct patient care, collective patient care , general tasks and other tasks [37]. The relation between the patient characteristics and care time was studied in step 3[36], nine of the fifteen patient characteristics turned out to be significantly related to care time. In addition to care time related to patient characteristics, it was also assumed that there would be a patient-related 'baseline care time'. When a patient is admitted to a ward, nurses spend a certain amount of care time on this patient e.g., time that is spent on handing out meals, having a chat or tidying up) , regardless of the patient characteristics that apply. The observational study also yielded how much time nurses spent on non-patient related activities (step 4).The objective modelled workload (step 7) is then calculated by comparing required nursing time (step 5) to allocated nursing time (step 6). The total required nursing time is the result of adding up care time related to patient characteristics, baseline patient related care time and time for non-patient related activities as follows:

$$(a1 * n1 + a2 * n2 + a..* n..+..) + (b * N) = x$$

$$Yreq = \frac{x}{(1 - z)}$$

$a1 = additional\ care\ time\ when\ patient\ characteristic\ 1\ applies\ (minutes)$

$n1 = number\ of\ patients\ in\ the\ dayshift\ for\ whom\ characteristic\ 1\ applies$

$b = baseline\ care\ time\ per\ patient\ (minutes)$

$N = number\ of\ patients\ admitted\ in\ the\ dayshift$

$x = amount\ of\ care\ time\ for\ patient\ related\ activities\ (minutes)$

$z = \%\ of\ time\ spent\ by\ nurses\ on\ non - patient\ related\ activities$

$$\boldsymbol{Yreq = total\ required\ nursing\ time\ (minutes)}$$

Both required and allocated nursing times were calculated with corrections for nurse proficiency. In the work sampling source data, care times were corrected directly for nurse proficiency[36]. These corrected care times were the basis for calculating the require nursing time.
Allocated nursing time was determined by adding up the amount of nurses in the shift, multiplying this by the shift time and correcting for nurse proficiency. Proficiency was estimated by expert nurses. In a mini Delphi study, all six ward managers (all nurses with more than fifteen years of experience) of the wards involved in the study were asked to define nurse categories and corresponding proficiency levels. The mini Delphi consisted of two rounds and two sessions to discuss results. The Delphi yielded six nurse proficiency categories. The reference category is the fully qualified and experienced registered nurse: this was defined as the standard and set to a proficiency percentage of 100%. The proficiencies of the other five types of nurses (novice registered nurses, fulltime student nurses in their first or second year, fulltime student nurses in their third or fourth year, working student nurses in their first or second year, working student nurses in their third or fourth year) were offset against this standard.
The allocated nursing time is calculated as follows:

$$(m1 * p1 + m2 * p2 + m.. * p.. + ..) = Yall$$

$$m1 = amount\ of\ time\ nurse\ 1\ was\ observed\ in\ the\ day\ shift\ (minutes)$$

$$p1 = proficiency\ of\ nurse\ 1$$

$$\boldsymbol{Yall = total\ allocated\ nursing\ time\ (minutes)}$$

The modelled workload was calculated by dividing required nursing time by allocated nursing time. This ratio gives an objective indication of nurses' workload (W). W = 100% reflects that the allocated nursing time is perfectly balanced to the required nursing time; W<100% indicates overstaffing and W>100% understaffing.

$$W = 100\% * \frac{Yreq}{Yall}$$

$\boldsymbol{Yreq = total\ required\ nursing\ time\ (minutes)}$

$\boldsymbol{Yall = total\ allocated\ nursing\ time\ (minutes)}$

$\boldsymbol{W = modelled\ workload}\ (\%)$

This modelled workload was calculated retrospectively for each ward on each of the day shifts during the time study period.

Covariates: personal resources, job resources and job demands

Several covariates are included in the analysis, in order to discover potential interventions for balancing perceived workload in daily practice.

Personal resources were not expected to vary on a daily basis and were measured once at baseline, three weeks prior to the work sampling period. Personal resource proficiency was estimated by expert nurses, as mentioned above. Personal resource self-efficacy was measured by a scale based on Schwarzer & Jerusalem's general self-efficacy scale [59]. The scale contains five questions: 'When difficult problems occur at work, I know how to deal with them', 'At work, I reach my goal, even when unexpected situations occur', 'If I encounter obstacles at work, I always find a way around them', 'Even if it takes a lot of my time and energy, at work I achieve what I want' and 'If I encounter something new at work, I always know how to deal with it'. All questions had a five point answer scale, ranging from 'Agree completely' (5) to 'Disagree completely' (1). Scores on the five questions were summed to a total score and then averaged per respondent.

Job resources support from management and support from colleagues were measured on a daily basis for all nurses on duty during the time study period. The questions originated from the Questionnaire on the Experience and Evaluation of Work (QEEW [30]). Support from management was measured by the question 'I could count on my supervisor when I came across difficulties in my work today'. Support from colleagues was determined by the question 'I could count on my colleagues when I came across difficulties in my work today'. Both questions had a 5 point answer scale, ranging from 'Not at all' (1) to 'Very often' (5).

The job resource percentage of registered nurses on the ward was determined by mapping the nurse qualifications of all nurses involved in the study and calculating a percentage of registered nurses on duty on each ward for each day of the time study.

The job demands perceived equality of work distribution and perceived interruptions were also measured once at baseline. Perceived equality of work distribution was measured by the question 'Is the work distributed evenly across all employees of the department?'. Perceived interruptions

was determined by the question "Do you have to deal with interruptions in your work?". Both questions had a four point answer scale, ranging from 'Never' (1) to 'Always (4). These questions were derived from questionnaires often used by Dutch occupational health services.
The proportions of time spent on direct patient care and time spent on registration were derived from the observational study results [37] and determined per nurse per day. Direct patient care entails all activities that can be attributed to one specific patient, excluding registration. Registration is defined as all administrative and registration tasks done either on behalf of the patient (such as patient documentation, ordering medication and applying for tests and examinations) or as part of general administrative activities (such as ordering supplies, processing incoming mail).

Data analysis

A one-way ANOVA and Tukey post-hoc tests were performed to determine significant differences between wards for the modelled workload.
Linear mixed effects models [60] were used to study the relation between the modelled workload and perceived workload. Observations were not independent since the majority of nurses were observed more than once during the work sampling period (longitudinal data). Linear mixed effects models are suitable for analyzing longitudinal data. The modelled workload W was the independent variable and the five perceived workload measures were the outcome variables; each model included a random intercept per nurse to account for dependence of measures within nurses over time.
For each of the five perceived workload measures, the relation between the modelled workload W and the perceived workload was first tested, with the wards as fixed effects. Subsequently, the personal resources (2 variables), job resources (3 variables) and job demands (4 variables) were added to the model in blocks to test the direct effects on perceived workload. In the last step, interactions between the modelled workload estimate and the four job demands and three job resources were introduced, to test for moderation. This resulted in five estimated models for each of the five outcome measures.
The significance level was set to 0.01. Model fits were evaluated for each outcome measure by comparing the Bayesian Information Criterion (BIC) values. BIC was chosen over the Akaike information criterion and likelihood ratio testing because the models include a relatively large number of independent variables and BIC is more conservative when testing several parameters at once.

Ethical considerations

The study guaranteed the privacy of involved staff and patients. The study protocol was reviewed and approved by the medical ethical review board of the UMC Utrecht, protocol number 14-165/C.

Results

Baseline and daily questionnaires

The daily questionnaire was filled out 694 times, resulting in an average response rate of 58%. The baseline measure questionnaire was returned by 162 nurses; a response rate of 65%. Details on response rates, population characteristics and average responses for personal resources and job resources are shown per ward in **Table 1**.

Table 1: Response rates, population characteristics and average response of the respondents of the baseline and daily questionnaires

	Ward	1				2				3				4				5				6			
**Response**		n	%	Mean	SD	n	%			n	%			n	%			n	%			n	%		
**Baseline response**		**28**	**72%**			**33**	**73%**			**29**	**54%**			**31**	**62%**			**16**	**53%**			**25**	**83%**		
**Gender**	Male	2	7%			3	9%			5	17%			6	19%			0	0%			2	8%		
	Female	26	93%			30	91%			24	83%			25	81%			16	100%			23	92%		
**Age category**	<20	1	4%			0	0%			0	0%			0	0%			0	0%			1	4%		
	20-30	10	36%			15	45%			17	59%			21	68%			8	50%			13	52%		
	30-40	3	11%			9	27%			4	14%			3	10%			2	13%			3	12%		
	40-50	3	11%			4	12%			1	3%			1	3%			0	0%			2	8%		
	>=50	8	29%			2	6%			3	10%			2	6%			5	31%			5	20%		
	Unknown	3	11%			3	9%			4	14%			4	13%			1	6%			1	4%		
**Type of nurse**	Registered nurse	24	64%			31	70%			20	52%			21	58%			10	44%			16	48%		
	Student nurse	4	36%			2	30%			9	48%			10	42%			6	56%			9	52%		
**Self-efficacy (range 1-5)**				3,5	0,4			3,6	0,5			3,6	0,4			3,7	0,4			3,6	0,4			3,6	0,5
**Interruptions (range 1-4)**				2,5	0,8			2,7	0,7			2,3	0,7			2,3	0,8			2,4	0,8			2,0	0,9
**Equal work distribution (range 1-4)**				2,8	0,5			2,5	0,7			2,7	0,6			2,6	0,7			2,8	0,4			2,6	0,7
**Daily response**		**91**	**58%**			**107**	**45%**			**141**	**63%**			**151**	**66%**			**93**	**57%**			**111**	**61%**		
**Gender**	Male	15	16%			3	3%			36	26%			30	20%			8	9%			12	11%		
	Female	68	75%			96	90%			102	72%			95	63%			80	86%			91	82%		
	Unknown	8	9%			8	7%			3	2%			26	17%			5	5%			8	7%		
**Age category**	<20	3	3%			0	0%			0	0%			11	7%			4	4%			12	11%		
	20-30	49	54%			66	62%			106	75%			98	65%			62	67%			67	60%		
	30-40	8	9%			16	15%			12	9%			9	6%			19	20%			18	16%		
	40-50	13	14%			15	14%			8	6%			4	3%			0	0%			3	3%		
	>=50	10	11%			2	2%			12	9%			3	2%			3	3%			3	3%		
	Unknown	8	9%			8	7%			3	2%			26	17%			5	5%			8	7%		
**Type of nurse**	Registered nurse	62	68%			70	65%			68	48%			85	56%			51	55%			41	37%		
	Student nurse	29	32%			37	35%			73	52%			66	44%			42	45%			70	63%		
**Support management (range 1-5)**				3,8	1,1			3,7	1,2			4,1	1,1			3,9	1,0			3,9	1,1			4,1	1,0
**Support colleagues (range 1-5)**				4,1	0,8			4,0	0,9			4,2	0,9			4,0	0,9			4,2	0,8			4,3	0,8

Not surprisingly, the vast majority of nurses are female (**Table 1**). Of the respondents of the baseline questionnaire, between 44% and 70 % were registered nurses; in the daily questionnaires this ranged between 37% and 68%. Gender and age category could not always be ascertained, for example for students who left the hospital or for temporary nursing staff. 36% to 68% of respondents were between 20 and 30 years old. The relatively high proportion of young nurses can be explained by the study setting: the research took place in an academic hospital, with specific training and educational tasks. On all wards, support of colleagues scored higher on average than support of management (4.0 to 4.3 and 3.7 to 4.1, respectively). Self-efficacy seems quite stable across wards, with average scores ranging between 3.5 and 3.7 and standard deviations between 0.4 and 0.5.

Modelled workload

One of the main practical concerns in the study hospital was whether the workload was divided equally across wards. In order to answer this question, modelled workload was analyzed per ward. **Figure 2** presents the average modelled workload per day shift, calculated retrospectively for the days of the work sampling period. Each line represents one ward. During the work sampling period, on two day shifts equipment failed on a ward and on one day the data download failed for one ward. This meant that not all the required data were available to calculate the modelled workload on those days, so three day shifts were excluded for all wards. Days 1, 3 and 5 are missing. Since these failures occurred randomly, it is expected that the missing data do not influence the outcomes.

Figure 2: Average modelled workload per ward per day, calculated retrospectively for the work sampling period

The post hoc tests found that modelled workload on ward 6 was significantly lower than on wards 1 (estimated difference -32.3%, confidence interval -58.1% to -6.4%, p-value 0,001) and 4 (estimated difference -36.6%, confidence interval -62.4% to -10.7%, p-value <0,001), and workload on ward 3 was significantly lower than on ward 4 (estimated difference 30.2%, confidence interval -56.0% to -4.3%, p-value 0,002).

Perceived workload

For the perceived workload measures, data were available for all fifteen day shifts in the work sampling period.

Figure 3 presents the average perceived workload per ward per day shift, for each of the five perceived workload measures.

Figure 3: Five line graphs with average perceived workloads per ward per day for amount of work, work pace, mental load, emotional load and physical load

The graphs of amount of work and work pace are quite similar. The graph of emotional load is more stable and on average lower than the other measures. To a lesser extent, the same goes for the results on physical load. On day 4 on ward 2, workload was apparently perceived exceptionally high on all of the five outcomes measures. On day 9 on ward 2, all workloads were perceived to be relatively low, but mental load peaked.

Effects of personal resources, job resources and job demands

For all five perceived workload measures, the models with interactions did not perform better than the models without interactions, hence the interactions are not shown in Table 2.. This means that no moderation was found on the relation between modelled and perceived workload, all significant effects of variables were direct effects on perceived workload. Details on model fits can be found in **Appendix 1**.

For three outcome measures (mental load, emotional load and physical load), the models that included the job demands did not perform better than the models without the job demands. Therefore, no results are shown for the job demands for these three outcome measures.

Table 2: Effects of job demands, job resources and personal resources on five outcome measures of perceived workload (range 1 to 5)

		**Perceived Work pace**		**Perceived Amount of work**		**Perceived Mental load**		**Perceived Emotional load**		**Perceived Physical load**	
**Type of variable**	**Variable**	Estimated effect *	Standard error	Estimated effect *	Standard error	Estimated effect *	Standard error	Estimated effect *	Standard error	Estimated effect *	Standard error
**Job demand**	Modelled workload	**0.209**	**0.024**	**0.198**	**0.025**	**0.141**	**0.021**	**0.043**	**0.013**	**0.047**	**0.017**
**Personal resource**	Perceived self-efficacy	0.010	0.211	-0.03	0.248	-0.12	0.212	-0.08	0.147	-0.10	0.218
	Estimated nurse proficiency %	-0.00	0.039	-0.03	0.046	0.008	0.037	-0.00	0.026	-0.00	0.038
**Job resource**	Perceived support of management	-0.01	0.057	-0.05	0.061	-0.08	0.052	-0.02	0.032	-0.03	0.043
	Perceived support of colleagues	**-0.23**	**0.073**	**-0.30**	**0.079**	**-0.23**	**0.067**	**-0.11**	**0.042**	**-0.22**	**0.057**
	% of nurses on ward that is registered nurse	0.088	0.057	0.068	0.061	0.083	0.052	**0.085**	**0.032**	0.076	0.044
**Job demand**	% nursing time spent on direct patient care	**0.165**	**0.037**	**0.228**	**0.040**	.	.	.	.	.	.
	% nursing time spent on registration	-0.19	0.080	**-0.29**	**0.085**	.	.	.	.	.	.
	Perceived equality in work distribution	-0.10	0.171	-0.15	0.201	.	.	.	.	.	.
	Perceived interruptions	0.232	0.120	0.150	0.142	.	.	.	.	.	.

*For variables with ordinal scales (for example from "Never" to "Always", translated into 1 to 4 points), a rise of one full point results in the effect shown in Table 3. For ratio variables, a rise of 10% results in the effect shown in Table 3.

Significant effects are printed in bold.

The results indicate a positive correlation between all perceived workload measures and modelled workload. Every 10% increase in estimated workload (on a scale that ranges roughly between 70 and 170%) is associated with a 0.209 increase in perceived work pace (on a scale from 1 to 5 this is a 5.2% increase), 0.198 (5%) in perceived amount of work, 0.141 (3.5%) in perceived mental workload, 0.043 (1.1%) in perceived emotional workload and 0.047 (1.2%) in perceived physical load.

Neither of the personal resources were significantly related to any of the perceived workload measures. The scale for self-efficacy was tested for internal consistency by calculating Cronbach's alpha. Alpha was 0.657 and did not improve if items were deleted from the scale. Since this practical scale is used often by occupational health services, it was decided to keep the scale in the study and test whether it was of influence on perceived workload even though Cronbach's alpha was relatively low.

Support from management was not related to any of the perceived workload measures, whereas support from colleagues was negatively associated with all outcome measures. A 1-point increase (on an scale of 1 to 5) in experienced support from colleagues reduced the experienced work

pace with 0.23 (5.8%), amount of work with 0.30 (7.5%), mental workload with 0.23 (5.8%), emotional workload with 0.11 (2.8%) and physical load with 0.22 (5.5%).

The percentage of registered nurses on the ward is correlated with the perceived emotional workload, but not the other subjective workload outcomes. Every 10% increase in proportion of registered nurses on a ward results in 0.085 (2.1%) increase in experienced emotional workload. The proportion of time spent on direct patient care and the proportion of time spent on registration were significantly related to perceived workload. For each 10% increase in time spent on direct patient care, there was a 0.165 (4.1%) point rise in perceived work pace and 0.228 (5.7%) point rise in perceived amount of work. Every 10% increase in proportion of time spent on registration gave a 0.29 (7.3%) decline in perceived amount of work. The other job demands were not significantly related to any of perceived workload measures.

Proportional effects for significant variables are summarized in **Table 3**.

Table 3: Proportional effects for variables with significant effect on perceived workload

			Perceived Work pace	Perceived Amount of work	Perceived Mental load	Perceived Emotional load	Perceived Physical load
**Type of variable**	**Variable**	**Increase**	Estimated proportional	Estimated proportional	Estimated proportional	Estimated proportional	Estimated proportional
**Job demand**	Modelled workload	+10%	+5.2 %	+5 %	+3.5 %	+1.1 %	+1.2 %
**Job resource**	Perceived support of colleagues	+1 point	-5.8 %	-7.5 %	-5.8 %	-2.8 %	-5.5 %
	% of nurses on ward that is registered nurse	+10%				+2.1 %	
**Job demand**	% nursing time spent on direct patient care	+10%	+4.1 %	+5.7 %			
	% nursing time spent on registration	+10%		-7.3 %			

*Estimated proportional effect on the outcome variable (expressed in % of rise in points on a 1-5 scale) for a rise of either 10% or 1 point in the independent variable

Discussion

Findings

The modelled workload is significantly different between wards 6 and 1, between 6 and 4 and between 3 and 4. The estimated differences are quite large; -32.3%, -36.6% and -30.2% respectively but so are the corresponding confidence intervals: -58.1% to -6.4%, -62.4% to -10.7% and -56.0% to -4.3% respectively.

The first hypothesis was accepted, since there is a linear correlation between the modelled workload and all five perceived outcome measures. Every 10% increase in modelled workload results in a 0.209 (5.2%) increase in perceived work pace, 0.198 (5%) in perceived amount of work, 0.141 (3.5%) in perceived mental workload but only 0.043 (1.1%) in perceived emotional workload and 0.047 (1.2%) in perceived physical load. Apparently, modelled workload *W* has the biggest effect on work pace, amount of work and mental workload. This makes sense, considering that the modelled workload *W* is based on comparing required and allocated care time only, and does not give any insight in emotional and physical requirements. Having to work fast due to a lack of balance between required and allocated resources may result in an experienced higher mental load because tasks that need focus and concentration need to be done under time pressure. Modelled workload, as found in this study, varies roughly between 70% and 170%; a range of 100%. With every 10% increase of the modelled workload, the perceived work pace increases with 5.2% and the perceived amount of work with 5.0%. It seems that perceived workload does not rise to the same extent as modelled workload. However, we need to consider that the answer range that was provided for all perceived workload measures ranges from 1 to 5 on an ordinal scale, which is quite a narrow range. Respondents therefore had limited options to express their perceptions of workload. In future research, a broader range of response may be of value to get a more detailed insight on the extent to which modelled workload influences perceptions.

Of the perceived workload measures, work pace and amount of work show a similar pattern. In the QEEW [30] these two are brought together in one construct: work pressure. In our study, we made the decision to separate the two in order to see whether the covariates may have different effects on each measure. Indeed this turned out to be the case; percentage of time spent on registration was correlated with perceived amount of work but not with work pace. Apparently, researching these two measures as separate constructs pays off. The graph of emotional load is most stable and on average reports a lower workload than the other measures. To a lesser extent, the same goes for the results on physical load. There were two peak days for ward 2 (days 4 and 9), we could not retrieve what would have been the reason for this. Especially the peak in mental

workload on day 9 is puzzling, since all other workloads measures on that ward on that day were relatively low.

Personal resources self-efficacy and proficiency were not related to any of the outcome measures. As for self-efficacy, this is not in line with findings in other studies, such as those of Spence Laschinger [54] and Martens [56], where positive correlations were found between (occupational coping) self-efficacy and workload and stress of staff shortages. Our findings do correspond with Schmidt's[44], who did find an effect of self-efficacy on job strain but not on perceived workload. However, the internal consistency of the scales for self-efficacy used in our study turned out to be low. The questions in the scale are insufficiently correlated to be seen as one construct, so no definite conclusion can be drawn about the effect of self-efficacy on perceived workload. It is recommended that in further research, other validated scales for self-efficacy are used. A study on nurse risk assessment decisions [61], showed that under conditions without time pressure, nurses with clinical expertise (i.e. more proficient nurses) performed better than novice nurses; the positive effects of clinical expertise, however, were negated when time pressure was introduced to clinical simulations. Since proficiency of nurses was not significantly related to workload in our study and in addition the workload estimate W was higher than 100% in 4 out of 6 wards, which indicates understaffing, possibly, this effect also occurred in our study setting. There was no evidence that the personal resources are related to the perceived workload measures; no direct effects or moderation were found, so hypothesis two was rejected.

Of the job demands other than workload, only the proportions of direct patient care and registration were of significance. An increased proportion of direct patient care results in increased perceptions of work pace and amount of work. This may be explained by the fact that time spent with the patient is regarded as more demanding; possibly patient related tasks are considered more urgent than other tasks and add to perceived work pressures. In line with this finding, an increase in proportion of time spent on registration gave a decline in perceived amount of work. Registration might be regarded as a less demanding task which may influence the perception of the work pressure. More research is needed on this, but the results may be explained by the fact that registration takes place in a quiet environment, away from the patient and thus away from potential pressures of patients and relatives or other caregivers. Possibly administrative work can form a stable, quiet moment in the working day where nurses can focus on this one task, whilst sitting down at a desk, instead of having to hurry along between tasks in different patient rooms. Also, unexpectedly, perceived work interruptions seemed irrelevant to perceived workload. Based on the available literature [38, 40], which shows a negative correlation between interruptions and patient safety, for example when performing complicated tasks such as

medication preparation, a positive correlation between interruptions and perceived workload was expected, at least with outcome measure perceived mental load. Perceived equality in work distribution was also not proven to be correlated to any of the workload outcome measures. To our knowledge there is no available literature on the effects of equal work distribution in the day shift on perceived workload. There are studies on distributive organizational justice (which reflects the perceived fairness in decision outcomes, such as work scheduling), and these suggest a negative correlation between distributive justice and intention to leave [62] and a positive correlation between distributive justice and quality of work life (for example psychological well-being, workload and work satisfaction)[63]. Assuming that equal work distribution would be also perceived as a fair distribution, a positive correlation between equal work distribution and perceived workload was expected but not proven. However, upon studying the different model fits for all outcome measures, there was a notable finding. For outcome measure perceived mental workload, two models scored almost similar on the BIC (see **Appendix 1**, **Table 3**): the model with all personal and job resources and all job demands (BIC=1061.9) and the model that also included interactions (BIC=1062.1). The model with interactions showed a significant moderation on the relation between modelled and perceived workload, for job demands work interruptions and equality of work distribution.

The job resources support from colleagues and proportion of registered nurses were significantly related to perceived workload. Support from colleagues proved to be an important factor in workload perception, since it was negatively correlated to all outcome measures. This is in line with findings of other studies [7, 47, 48], where teamwork was shown to offset negative effects of high workload. The fact that the effect of teamwork was found to be less strong on outcome measure perceived emotional workload was unexpected; apparently support from colleagues is more important in handling workload in the cognitive and physical domain than when coping with emotional challenges. The percentage of registered nurses on the ward is positively correlated with the perceived emotional workload, although not very strong. It was expected that registered nurses would be better equipped to handle emotional stressors than student nurses, but apparently, there is more to this. Possibly, registered nurses feel more responsible for their work than student nurses, which may increase emotional load. Or maybe registered nurses are more likely to be given the more emotionally challenging tasks than student nurses. More research is needed to explain this effect. Perceived support from management did not turn out to be significantly related to any of the outcome measures. A study by MacPhee [14] did show that support of unit-level management in managing workload could have positive effects on perceived quality of care and job outcomes, but this study did not test the direct relation between support

of management and perceived workload. In the present study, the relation between support from management and workload was tested, but could not be proven. When considering which support is more important to nurses, apparently sticking together as a team is more important to how workload is perceived than getting support from ward leadership. This suggests that ward leadership should consider their role in enhancing teamwork and teambuilding on the ward.
For all outcome measures, the models without the interactions showed a better fit than the models with interactions. This means that there is only evidence for direct effects, there is no moderation, which means that hypotheses three and four must be rejected. More research is recommended on the effects of perceived interruptions and perceived equality of work distribution on perceived mental workload, because the model that included moderation was almost as good as the model without.

Strengths and limitations

This was a longitudinal, multilevel study, which is rare in this field of research. The fact that an objective (modelled) and subjective (perceived) workload measures were combined in one study is unique.
Another strong feature of this study was that nurses were in the lead and had complete freedom in determining a set of patient characteristics that they believed influence care time the most. This resulted in a new set of characteristics, several of which have not been studied before, such as a patient's bodily proportions and one-on-one care. The effects of the patient characteristics on care time were quantified by work sampling over a relatively long study period of 15 day shifts.
Nurses involved in the study stressed the importance of nurse proficiency in workload calculations and suggested a new way of defining nurse proficiency. Data were corrected for this newly defined nurse proficiency, which has not been done before and proved to be an important factor to include in the analysis. The proportion of students is relatively high in our study, since the study setting is an academic hospital and two of the wards in the study are also specific learning wards which have a higher proportion of students than usual. Correcting for nurse proficiency is especially relevant in a teaching hospital setting.
A new concept 'baseline care time' was defined and quantified by deriving estimated marginal means from the mixed model, which has not been described in literature before.
The combination of a modelled workload measure and five perceived workload measures in one study is unique. Also, testing for moderation of job demands and job resources on the relation between the modelled and perceived workloads has not been done before.
The overall response rates on the baseline measure questionnaire and the daily questionnaire that

was used during the work sampling period were 65% and 70% respectively, which is considered to be quite good [64].

There are several limitations that need to be addressed. This study was set in an academic hospital, which makes it uncertain whether the study results can be readily applied to different settings, such as general hospitals. Nurses' activities and the patient mix in general hospitals are likely to be different than in academic hospitals, if this study is to be applied in such a setting, the framework can be the same, but it is recommended to review the list of patient characteristics and to repeat the work sampling. The same goes for departments with a unique nature, such as pediatric and psychiatric wards.

The confidence intervals for the estimated differences of modelled workload between wards were relatively high. For future research, a larger sample size is recommended in order to make a more accurate estimate of differences between wards.

The scales used to measure self-efficacy were chosen for practical reasons and were not validated by means of international publications. The items on the scale proved not to be internally consistent; future research should include validated scales of self-efficacy. Since the items do not represent one construct, no conclusion can be drawn about the effect of self-efficacy on perceived workload.

Perceived equality of work distribution and perceived interruptions were both measured by one question. Possibly, the use of more extensive measures would have influenced results. More research is recommended on this.

In this study, proficiency was estimated by a mini-Delphi study among head nurses. Another way to measure the proficiency of nurses would be to keep track of the actual exact time spent on each activity, calculate an estimate per activity per type of nurse and derive the proficiency percentage from these estimates. However, since there were 6 types of nurses and 24 activity groups in the study, this approach would have required a much larger sample size and a more accurate measurement of time spent on an activity than work sampling every ten minutes. For practical reasons (costs, registration), this was not possible, and the choice was made to estimate nurse proficiency instead.

Lastly, the work sampling study was done during fifteen consecutive dayshifts, which is quite a long time period but also only one time period. Possibly, results would have been more powerful if the study was done over two separate time periods. However, due to practical issues we did not chose this option. If the study was set over two time periods, staff would need to be trained and informed twice (for example not all medical students would be available for both time periods

and new nursing students would have entered the wards), equipment would need to be rented twice and the preparations such as barcoding patient rooms would need to be done again. This would have become too costly so the study team opted against this.

Concluding remarks

The study presents a method to calculate an objective measure for workload of nurses, which is positively associated with the perceived workload. This modelled workload can be used to detect differences in workload between wards, which may be useful to more evenly distribute workload in order prevent issues in the domain of e.g. organizational justice. When sufficient historical data are collected, an average patient characteristics profile per admission day can be composed for treatments such as operations. This way, an indication of workload of nurses on surgical hospital wards can be predicted based on operating room schedules. Besides the modelled workload, job resources support of colleagues and job demands time spent on direct patient care and time spent on registration had the biggest significant effects on perceived workload. In times when workload is high, extra effort in teambuilding is likely to have a positive effect on perceived workload. In these circumstances, unit-level management should consider focusing their attention on facilitating the nursing team to work together smoothly and on enhancing team spirit. Since time spent on direct patient care is positively associated with perceived workload and time spent on registration is negatively associated with perceived workload, a good balance between time spent on direct patient care and registration may also benefit perceptions of workload.

The findings of this research can help nurse management in allocating resources and directing their attention to the most relevant factors so workload of nurses is better balanced, which in turn leads to a higher quality of care, keeping nurses healthy and the prevention of additional costs for overstaffing, absenteeism or high turnover of nurses.

References

1. Cuckler GA, Sisko AM, Poisal JA, Keehan SP, Smith SD, Madison AJ, Wolfe CJ, Hardesty JC: **National Health Expenditure Projections, 2017-26: Despite Uncertainty, Fundamentals Primarily Drive Spending Growth.** In: *Health Affairs.* vol. 37, 1 March 2018 edn; 2018.
2. Martín Martín JJ, Puerto López del Amo González M, Dolores Cano García M: **Review of the literature on the determinants of healthcare expenditure**. *Journal of applied economics* 2011, **43**(1):19-46.
3. Dall TM, Gallo PD, Chakrabarti R, West T, Semilla AP: **An Aging Population And Growing Disease Burden Will Require ALarge And Specialized Health Care Workforce By 2025**. *Health affairs* 2013, **32**(11).
4. Zhang X, Tai D, Pforsich H, Lin VW: **United States Registered Nurse Workforce Report Card and Shortage Forecast: A Revisit.** *American Journal of Medical Quality* 2017, **33**(3):229-236.
5. WorldHealthOrganization: **Global Strategy on Human Resources for Health: Workforce 2030**. In. Edited by Europe WHORof: World Health Organization; 2014.
6. European Commission: **Second Biennial Report on social services of general interest**. In. Edited by Directorate-General for Employment Social Affairs and Inclusion; 2011.
7. Van Bogaert P, Peremans L, Van Heusden D, Verspuy M, Kureckova V, Van de Cruys Z, Franck E: **Predictors of burnout, work engagement and nurse reported job outcomes and quality of care: a mixed method study**. *BMC Nurs* 2017, **16**:5.
8. Yu D, Ma Y, Sun Q, Lu G, Xu P: **A nursing care classification system for assessing workload and determining optimal nurse staffing in a teaching hospital in China: A pre-post intervention study**. *International journal of nursing practice* 2015, **21**(4):339-349.
9. Aiken LH: **Nursing staff and education and hospital mortality in nine European countries: a retrospective observational study**. *Lancet* 2014, **383**(9931):1824-1830.
10. Liu LF, Lee S, Chia PF, Chi SC, Yin YC: **Exploring the association between nurse workload and nurse-sensitive patient safety outcome indicators**. *The journal of nursing research : JNR* 2012, **20**(4):300-309.
11. Unruh L: **Nurse staffing and patient, nurse, and financial outcomes**. *The American journal of nursing* 2008, **108**(1):62-71; quiz 72.
12. Hinno S, Partanen P, Vehvilainen-Julkunen K: **Nursing activities, nurse staffing and adverse patient outcomes as perceived by hospital nurses**. *J Clin Nurs* 2012, **21**(11-12):1584-1593.
13. Källberg AS, Ehrenberg A, Florin J, Östergren J, Göransson KE: **Physicians' and nurses' perceptions of patient safety risks in the emergency department.** *International emergency nursing* 2017, **33**:14-19.
14. MacPhee M, Dahinten VS, Havaei F: **The Impact of Heavy Perceived Nurse Workloads on Patient and Nurse Outcomes**. *Administrative Sciences* 2017, **7**:1-17.
15. Van Bogaert P, Clarke S, Willems R, Mondelaers M: **Staff engagement as a target for managing work environments in psychiatric hospitals: implications for workforce stability and quality of care**. *J Clin Nurs* 2013, **22**(11-12):1717-1728.
16. Aiken LH, Sermeus W, Van den Heede K, Smith HL, Kutney-Lee A: **Patient safety, satisfaction, and quality of hospital care: cross sectional surveys of nurses and patients in 12 countries in Europe and the United States.** *BMJ* 2012, **344**(E1717).

17. Bakker A.B. DE, Verbeke W.: **Using the Job Demands-Resources model to predict burnout and performance**. *Human Resource Management* 2004, **43**:83-104.
18. Spence Laschinger HK, Grau AL, Finegan J, Wilk P: **Predictors of new graduate nurses' workspace well-being: testing the job demands-resources model**. *Health care management review* 2012, **37**(2):175-186.
19. Ohue T, Moriyama M, Nakaya T: **Examination of a cognitive model of stress, burnout, and intention to resign for Japanese nurses**. *Japan journal of nursing science : JJNS* 2011, **8**(1):76-86.
20. Toh SG, Ang E, Devi MK: **Systematic review on the relationship between the nursing shortage and job satisfaction, stress and burnout levels among nurses in oncology/haematology settings**. *International journal of evidence-based healthcare* 2012, **10**(2):126-141.
21. Mudaly P, Nkosi ZZ: **Factors influencing nurse absenteeism in a general hospital in Durban, South Africa**. *J Nurs Manag* 2015, **23**(5):623-631.
22. Van Bogaert P, van Heusden D, Timmermans O, Franck E: **Nurse work engagement impacts job outcome and nurse-assessed quality of care: model testing with nurse practice environment and nurse work characteristics as predictors**. *Frontiers in psychology* 2014, **5**:1261.
23. Lacey SR, Cox KS, Lorfing KC, Teasley SL, Carroll CA, Sexton K: **Nursing support, workload, and intent to stay in Magnet, Magnet-aspiring, and non-Magnet hospitals**. *The Journal of nursing administration* 2007, **37**(4):199-205.
24. Leone C, Bruyneel L, Anderson JE, Murrells T, Dussault G, Henriques de Jesus E, Sermeus W, Aiken L, Rafferty AM: **Work environment issues and intention-to-leave in Portuguese nurses: A cross-sectional study**. *Health Policy* 2015, **119**(12):1584-1592.
25. Twigg D, Duffield C: **A review of workload measures: a context for a new staffing methodology in Western Australia**. *Int J Nurs Stud* 2009, **46**(1):131-139.
26. Jones TL, Hamilton P, Murry N: **Unfinished nursing care, missed care, and implicitly rationed care: State of the science review.** *International journal of nursing studies* 2015, **52**:1121-1137.
27. Myny D, Van Goubergen D, Gobert M, Vanderwee K, Van Hecke A, Defloor T: **Non-direct patient care factors influencing nursing workload: a review of the literature**. *J Adv Nurs* 2011, **67**(10):2109-2129.
28. Alghamdi MG: **Nursing workload: a concept analysis**. *Journal of nursing management* 2016, **24**(4):449-457.
29. Holden RJ, Scanlon MC, Patel NR, Kaushal R, Escoto KH, Brown RL, Alper SJ, Arnold JM, Shalaby TM, Murkowski K *et al*: **A human factors framework and study of the effect of nursing workload on patient safety and employee quality of working life**. *BMJ quality & safety* 2011, **20**(1):15-24.
30. Van Veldhoven M, Meijman TF: **Het meten van psychosociale arbeidsbelasting met een vragenlijst: de Vragenlijst Beleving en Beoordeling van de Arbeid [The measurement of psychosocial job demands with a questionnaire: the questionnaire on perception and evaluation of the work],**. In. Edited by Arbeidsomstandigheden NIv. Amsterdam; 1994.
31. Demerouti E, Bakker AB, Nachreiner F, Schaufeli WB: **The job demands-resources model of burnout**. *The Journal of applied psychology* 2001, **86**(3):499-512.

32. Bakker AB, Demerouti E: **Job demands-resources theory: Taking stock and looking forward**. *J Occup Health Psychol* 2017, **22**(3):273-285.
33. Schaufeli WB, Bakker AB: **Job demands, job resources, and their relationship with burnout and engagement: a multi-sample study**. *Journal of organizational behaviour* 2004(25):293-315.
34. Oetelaar van den WFJM, Stel van HJ, Rhenen van W, Stellato RK, Grolman W: **Balancing nurses' workload in hospital wards: study protocol of developing a method to manage workload**. *BMJ Open* 2016, **6**(11).
35. Van den Oetelaar WFJM, van Stel HF, van Rhenen W, Grolman W: **Identifying patient characteristics related to care time: a Delphi study in surgical hospital wards**. *Submitted for Review* 2017.
36. Oetelaar van den WFJM, Rhenen van W, Stellato RK, Grolman W: **Balancing workload of nurses: Linear mixed effects modelling to estimate required nursing time on surgical wards**. *Nursing Open* 2019, **7**(1):235-245.
37. van den Oetelaar WFJM, Stel van HJ, Stellato RK, van Rhenen W, Grolman W: **Mapping nurses' activities in surgical hospital wards: a time study**. *PLOS ONE* 2017.
38. McGillis Hall L, Pedersen C, Fairley L: **Losing the Moment. Understanding Interruptions to Nurses' Work**. *The Journal of nursing administration* 2010, **40**(4):169-176.
39. Duffield C, Diers D, O'Brien-Pallas L, Aisbett C, Roche M, King M, Aisbett K: **Nursing staffing, nursing workload, the work environment and patient outcomes**. *Appl Nurs Res* 2011, **24**(4):244-255.
40. Redding D, A., Robinson S: **Interruptions and Geographic Challenges to Nurses' Cognitive Workload**. *Journal of nursing care quality* 2009, **24**(3):194–200.
41. Myny D, Van Hecke A, De Bacquer D, Verhaeghe S, Gobert M, Defloor T, Van Goubergen D: **Determining a set of measurable and relevant factors affecting nursing workload in the acute care hospital setting: a cross-sectional study**. *Int J Nurs Stud* 2012, **49**(4):427-436.
42. Khademi M, Mohammadi E, Vanaki Z: **Resources–tasks imbalance: Experiences of nurses from factors influencing workload to increase**. *Iranian Journal of Nursing and Midwifery Research* 2015, **20**(4):476–483.
43. Bakker ABD, E; Euwema M.C.: **Job resources buffer the impact of job demands on burnout**. *Journal of Occupational Health Psychology* 2005(10):170-180.
44. Schmidt KH, Distel S: **Job demands and personal resources in their relations to indicators of job strain among nurses for older people**. *Journal of Advanced Nursing* 2012, **69**(10):2185–2195.
45. Schaufeli WB, Bakker AB, Van Rhenen W: **How changes in job demands and resources predict burnout, work engagement, and sickness absenteeism**. *Journal of organizational behavior* 2009, **30**:893–917.
46. Bakker AB: **Job resources boost work engagement, particularly when job demands are high**. *Journal of Educational Psychology* 2007, **99**(2):274-284.
47. Yanchus NJ, Ohler L, Crowe E, Teclaw R, Osatuke K: **You just can't do it all': a secondary analysis of nurses' perceptions of teamwork, staffing and workload**. *Journal of Research in Nursing* 2017, **22**(4):313-325.
48. Sexton JB, Holzmueller CG, Pronovost PJ, Thomas EJ, McFerran S, Nunes J, Thompson DA, Knight AP, Penning DH, Fox HE: **Variation in caregiver perceptions of**

teamwork climate in labor and delivery units. *Journal of perinatology* 2006, **26**(8):463-470.

49. Van Bogaert P, Kowalski C, Weeks SM, Van Heusden D, Clarke SP: **The relationship between nurse practice environment, nurse work characteristics, burnout and job outcome and quality of nursing care: a cross-sectional survey**. *Int J Nurs Stud* 2013, **50**(12):1667-1677.
50. Duffield C, Roche M, Merrick ET: **Methods of measuring nursing workload in Australia**. *Collegian* 2006, **13**(1):16-22.
51. Tourangeau A: **Nursing skill mix and experience reduce patient mortality**. *Hospital quarterly* 2002, **5**(3):19-20.
52. Morris R, MacNeela P, Scott A, Treacy P, Hyde A: **Reconsidering the conceptualization of nursing workload: literature review**. *J Adv Nurs* 2007, **57**(5):463-471.
53. Hegney D, Plank A, Parker V: **Nursing workloads: the results of a study of Queensland Nurses**. *Journal of nursing management* 2003, **11**(5):307-314.
54. Spence Laschinger HK, Borgogni L, Consiglio C, Read E: **The effects of authentic leadership, six areas of worklife, and occupational coping self-efficacy on new graduate nurses' burnout and mental health: A cross-sectional study**. *International journal of nursing studies* 2015, **52**:1080–1089.
55. Pisanti R, Lombardo C, Lucidi F, Lazzari D, Bertini M: **Development and validation of a brief Occupational Coping Self-Efficacy Questionnaire for Nurses**. *Journal of advanced nursing* 2008, **62**(2):238-247.
56. Martens ML: **A Comparison of Stress Factors in Home and Inpatient Hospice Nurses**. *Journal of hospice and palliative nursing* 2009, **11**(3):144-153.
57. Brunetto Y, Rodwell J, Shacklock K, Farr-Wharton R, Demir D: **The impact of individual and organizational resources on nurse outcomes and intent to quit**. *Journal of Advanced Nursing* 2016, **72**(12):3093-3103.
58. McHugh MD, Lake ET: **Understanding clinical expertise: nurse education, experience, and the hospital context**. *Research in nursing & health* 2010, **33**(4):276-287.
59. Schwarzer R, Jerusalem M: **Generalized Self-Efficacy scale**. In: *In J Weinman, S Wright, & M Johnston, Measures in health psychology: A user's portfolio Causal and control beliefs* Windsor, UK: NFER - Nelson; 1995: 35-37.
60. Hedeker D, Gibbons RD: **Longitudinal data analysis**, 1st edition edn: Wiley-Interscience; 2006.
61. Thompson C, Dalgleish L, Bucknall T, Estabrooks C, Hutchinson AM, Fraser K, Vos De R, Binnekade J, Barrett G, Saunders J: **The effects of time pressure and experience on nurses' risk assessment decisions: A signal detection analysis.** . *Nursing Research* 2008(57):302-311.
62. Nelson MF, Tarpey RJ: **Work scheduling satisfaction and work life balance of nurses: the perception of organizational justice**. *Academy of Health Care Management Journal* 2010, **6**(1):25-36.
63. Gillet N, Fouquereau E, Bonnaud-Antignac A, Mokounkolo R, Colombat P: **The mediating role of organizational justice in the relationship between transformational leadership and nurses' quality of work life: A cross-sectional questionnaire survey**. *International journal of nursing studies* 2013, **50**:1359–1367.

64. Baruch Y: **Response Rate in Academic Studies—A Comparative Analysis**. *Human Relations* 1999, **52**(4):421-438.

Revised manuscript incl Track changes
Click here to access/download;Manuscript;Manuscript 5 relation obj subj job dem 20200319.docx

Formatted: Numbering: Continuous

Abstract

Aim

Calculating an objective, modelled workload, exploring the relation between this modelled workload and workload as perceived by nurses and studying the effects of certain job demands, job resources and personal resources.

Design

Academic hospital in the Netherlands. Six surgical wards, capacity 15-30 beds. Data collected over 15 consecutive day shifts.

Methods

Modelled workload is calculated as a ratio of required care time, based on patient characteristics, baseline care time and time for non-patient related activities, and allocated care time, based on the amount of available nurses. Both required and allocated care time are corrected for nurse proficiency. Five dimensions of perceived workload were determined by questionnaires. Both the modelled and the perceived workloads were measured on a daily basis. Linear mixed effects models study the longitudinal relation between this modelled and workload as perceived by nurses and the effects of personal resources, job resources and job demands. ANOVA and post-hoc tests were used to identify differences in modelled workload between wards.

Results

Modelled workload varies roughly between 70 and 170%. Significant differences in modelled workload between wards were found but confidence intervals were wide. Modelled workload is positively associated with all five perceived workload measures (work pace, amount of work, mental load, emotional load, physical load). In addition to modelled workload, the job resource support of colleagues and job demands time spent on direct patient care and time spent on registration had the biggest significant effects on perceived workload.

Conclusions

The modelled workload can be used to detect differences in workload between wards, which may be useful in distributing workload more evenly in order prevent issues in the domain of e.g. organizational justice. Average patient characteristic profiles per admission day can be composed

for treatments such as operations to predict an indication for workload. Extra effort in team building is likely to have a positive effect on perceived workload. A good balance between time spent on direct patient care and registration may also benefit perceptions of workload. The findings of this research can help nursing management in allocating resources and directing their attention to the most relevant factors for balancing workload.

Introduction

In healthcare, there is an increasing pressure on maintaining a high quality of care whilst containing expenditure [1]. Healthcare expenditure is increasing due to factors such as technological progress and an aging population with more chronic conditions [2, 3]. Healthcare providers are expected to maintain a high quality of care under increasing demand, with the same or less funding [4-6]. There is a risk that under these circumstances, nurses' workload will increase to alarming levels. Workload is considered unacceptable, if nurses are not able to meet patients' needs, physical as well as emotional [7] or if nurses' health is at risk. There is overwhelming evidence of the adverse effects of high workload of nurses. There is a direct relation between nurses' workload and patient satisfaction [8], patient outcomes [9-14] and nurse reported quality of care and performance [15-17]. High workload is also a predictor for nurses' job dissatisfaction, stress, burnout [18-20] and absenteeism [21], and has generally been shown to have a negative effect on job outcomes [22] and nurses' intention to leave [23, 24]. High turnover of nursing staff results in higher costs for training of new nurses, or hiring of temporary staff [11, 25]. Nurses also report that when workload is high, they cannot deliver all care that they believe they should [26]. Important tasks, such as tending to patients' emotional and psychological needs, are left undone, which leaves nurses feeling unsatisfied with their job and occupation, which in turn leads to an increasing intent to leave, lower reported quality of care and deteriorating patient satisfaction [26].

Formatted: Space After: 0 pt, Don't suppress line numbers

~~Studies have found negative associations between the workload of nurses and patient outcomes~~ [9, 14]~~, nurse engagement~~ [27-31]~~ and job outcomes~~ [22]~~, and positive relations with nurse burnout~~ [7, 18, 19]~~ and nurses' intention to leave the job~~ [23, 24]~~.~~ Managing ~~the~~ workload will therefore become increasingly important in preventing the vicious cycle where nurses ~~from~~are leaving the job, ~~which would in turn lead~~ which leads to a greater shortage and an even higher workload.

Despite the fact that a high workload can have such far-reaching practical implications, the concept of workload is not always clearly defined in research. It is often measured by using crude staffing ratios or determined by questionnaires regarding perceived workload. Also, many studies examine only one dimension of workload, such as perceived mental load or amount of work [32]. Alghamdi [33] defined workload as the amount of time and care that a nurse can devote (directly and indirectly) towards patients, workplace and professional development. This definition covers direct and indirect patient care but also non-nursing activities such as meetings and attending seminars. Alghamdi advocated a holistic approach and determined five defining attributes of

Formatted: Space After: 10 pt

nursing workload: the amount of nursing time spent on nursing care known as patient acuity; the level of nursing competency; the weight of nursing intensity (direct patient care); all the physical, mental and emotional efforts; and the ability of the nurse to change the plan (complexity of care). Holden [34] describes three different types of perceived workload: task-level, job-level and unit-level workload. Unit-level workload considers the balance between patient acuity and staffing, job-level workload entails general and specific demands of the job such as the general amount and difficulty of the work and the amount of concentration required to do it, and task-level workload relates to the demands and resources for a specific task such as medication preparation. These workload types describe different dimensions of workload and each type of workload has a specific effect on burnout, job dissatisfaction and the likelihood of medication errors. Holden's study did not consider emotional and physical workload, but recommended also taking these into account. In the Netherlands, the Questionnaire on the Experience and Evaluation of Work (QEEW [35]), an evidence based questionnaire, is widely used by Dutch occupational health services to measure the (psychosocial) working environment. It defines four types of workload: work pressure (a combination of work pace and amount of work), emotional load, mental load and physical effort.
The well-known Job Demands and Resources (JDR) model [36] also considers workload. The JDR model describes a health impairment process where high job demands lead to exhaustion and burnout. Job demands are those aspects of a job that require effort. On the other hand, the JDR model describes a motivational process where job resources play a crucial role. Job resources are functional in achieving work goals, reduce job demands and the associated physiological and psychological costs and stimulate personal growth and development[36]. Workload is considered a job demand in the JDR model; prolonged periods of high workload can lead to burnout, especially if there are insufficient job resources to counterbalance the effects of the job demands [27, 36, 37].

A previous study by our group [38] presented a method to calculate and predict nurses' workload by means of objective measures. Subsequent studies ~~[38].~~yielded a set of patient characteristics - developed by expert nurses- that were expected to be significantly related to care time [~~39, 40]~~.We determined significance and care time estimates of these characteristics [40] and in addition we also specified how much time is spent on patients regardless of these characteristics [40] and how much time is spent on non-patient related activities [41]. The importance of including nurse proficiency in workload calculations was also demonstrated [40]. ~~[38] Literature describes several methods to determine workload or nursing intensity [42-45], but none complied to all the demands set by this study team. The objective was to develop a practical tool to predict nurses'~~

~~workload that is user-friendly (easy to interpret and requiring limited additional registration); can be applied to different types of hospital wards; covers all activities of nurses (not only those activities that are directly patient-related); is suitable for prospective planning purposes; and takes into account nurses' proficiency (the level of education and work experience).~~

In the study presented in this paper, we build on the findings of this previous research. ~~and propose a method to calculate an objective measure for nurses' workload~~. ~~Balancing this~~ Both objective (calculated, modelled) and subjective (perceived, self-reported) workload measures are included in the study.

~~objective (modelled) workload is expected to also balance workload perceptions~~. The aim ~~of the current study~~ is to understand the relation between the objective, modelled workload and the workload as it is perceived by nurses, and to test the effects of several relevant factors on this relation. ~~To the best of our knowledge, studying the relation between objective and subjective workload, especially in a longitudinal setting, is unique in this field of research.~~ To the best of our knowledge, studying the relation between objective and subjective workload, especially in a longitudinal setting, is unique in this field of research.

~~The perceived workload is reported to be dependent on the ratio of required to allocated nursing times, but other factors are also known to be of influence. Several other variables will be included in the analysis, in order to discover potential interventions for balancing perceived workload in daily practice. Factors that consider employees other than nurses (such as nurse-physician relationships, support from logistic teams), factors that cannot be influenced by nurse management of the ward (such as social support at home) or factors that require major investments (such as ward layout and number of single rooms in a ward) were not included in the scope.~~

~~The well-known Job Demands and Resources (JDR) model~~ [36] ~~is chosen as a framework. The JDR model describes a health impairment process where high job demands lead to exhaustion and burnout. Job demands are those aspects of a job that require effort. Workload is considered a job demand in the JDR model.~~

This paper considers the effects of four ~~other~~ job demands, three job resources and two personal resources on the relation between modelled and perceived workload.

Perceived interruptions, perceived equal work distribution, time spent on registration and time spent on direct patient care are considered job demands in the context of this study. Dutch occupational health surveys, used by occupational health and safety services for preventive medicine purposes, often include questions on work interruptions and equal work distribution.

There is evidence that strongly relates the number of work interruptions to patient outcomes [46, 47] and nurses' workload [45, 48]. Equal work distribution has not been related to perceived workload in literature before. Registration required by the government, insurance companies and hospital management, is one of the reasons nurses experience a high workload. Myny [45] found a link between perceived workload and the number of mandatory registrations. Van Bogaert [7] also identifies a growing problem of additional registration. There are also indications that increased administration burden competes with the time intended to be spent on patient care; Khademi [49] stated that one of the important sources of increased nursing workload was an overemphasis of managers on frequent report writing, which competed with patient care delivery. Workload may also be experienced differently if the proportion of time that nurses can spend on direct patient care is low, due to either increased registration or other reasons.

The JDR model ~~also~~ postulates that the health impairment process can be mitigated by job resources and personal resources[36] . These resources help to achieve goals and stimulate personal development, resulting in intrinsic motivation and engagement [27, 28, 36, 50-52]. The present study will include three job resources: support from colleagues, support from management, and the proportion of registered nurses on the ward. Yanchus [53] found that the job resource teamwork (i.e, colleagues helping and backing each other up) counterbalances the effects of understaffing and high workload. Similarly, Sexton's research [54] found that workload pressures can be offset by a positive nursing team environment on a unit. This indicates that teamwork is an important factor in perceived workload. Van Bogaert et al. [7] found the same results in their study on the predictors of burnout, work engagement, nurse reported job outcomes and quality of care. Van Bogaert et.al. [55] also reported that hospital management directly influenced nurses' perceived workload. In MacPhee's study on the impact of heavy perceived workload of nurses on patient and nurse outcomes [14], it was noted that unit level leaders in particular could influence perceived quality of care and job outcomes by monitoring and responding to workload demands. In addition, there is much evidence that a nursing staff mix with a large proportion of registered nurses results in better patient outcomes [47, 56, 57]. The effect of the skill mix of nurses on perceived workload has not been extensively studied, although there is evidence that skill mix is an important factor when considering workload [58] and that a lower proportion of registered nurses on a ward is associated with increased workload [59]. In the current paper, the proportion of registered nurses on the ward is included and regarded as a job resource.

Two personal resources have been included in the current study: self-efficacy and ~~nurse~~ proficiency. Spence Laschinger [60] found a significant correlation between workload (as one of

the areas of work life) and occupational coping self-efficacy, defined as the self-appraisal of one's capability to cope with occupational burden in the workplace [61]. When the areas of work life, such as workload, are balanced, this has a positive effect on occupational coping self-efficacy. In the hospice setting, the stress of staff shortages decreased with increasing self-efficacy [62]. Schmidt et al. [51] found that self-efficacy had a direct effect on job strain in nursing homes, but they did not find evidence for an interaction between self-efficacy and perceived workload. Brunetto [63] found that self-efficacy reduced the effects of stress and enhanced job satisfaction, and herewith reduced nurses' intentions to leave the job; however, they did not study the effects on perceived workload.

Nurses' education and experience are related to clinical expertise [64]. A well-educated, experienced nurse may be able to handle workload better than a novice nurse. This study will test whether nurses with more working experience and who are considered to be more proficient experience workload differently than other nurses.

~~In this paper, five different measures of perceived workload are included: work pace, amount of work, mental workload, emotional workload and physical workload. The job demands (interruptions, work distribution, time for registration, time for direct patient care) and job resources (support from colleagues, support from management, the proportion of registered nurses on the ward) are included in the analyses as potential effect modifiers of the relation between modelled and perceived workload. Effects will be explored separately for each outcome measure. **Figure 1** presents a visualization of the model to be studied.~~

Field Code Changed

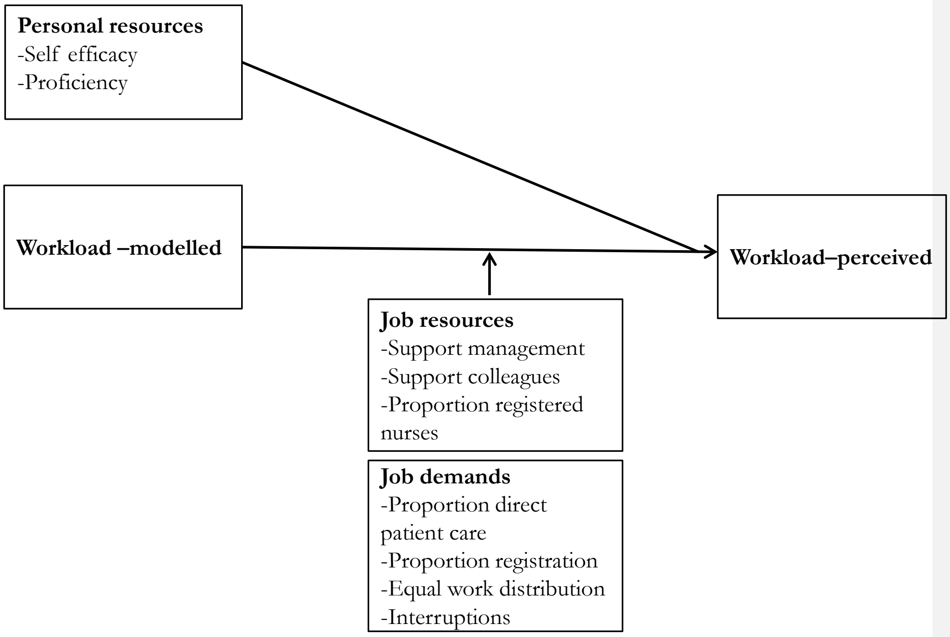

~~**Figure 1: Studying the relation between modelled and perceived workload (our hypotheses)**~~

We will test the following hypotheses:

Hypothesis 1: there is a correlation between the modelled (objective) workload measure and perceived (subjective) workload

Hypothesis 2: perceived workload is lower when personal resources self-efficacy and nurse proficiency are higher

Hypothesis 3: the job resources support from management, support from colleagues and proportion of registered nurses moderate the relation between modelled and perceived workload

Hypothesis 4: the job demands proportion of direct patient care, proportion of administration, work interruptions and perceived equality of work distribution moderate the relation between modelled and perceived workload.

~~To the best of our knowledge, studying the relation between objective and subjective workload, especially in a longitudinal setting, is unique in this field of research.~~

Method

Study ~~population and~~ setting

The research took place in an academic hospital in the Netherlands (private hospital). Six surgical wards were included (2 wards with 15 beds, 4 wards with 30 beds). The study focused on the day shift workload, because this is the shift during which the most nurses are required and most nursing activities are performed, some of which only occur in the dayshift.

Weekends were excluded because the task mix and staffing are very different in weekends and cannot be compared to the day shifts of regular weekdays.

Participants

All ~~R~~registered nurses and nurse students working on the study wards were included in the study.. Ward managers were excluded because they do not perform patient-related activities. The study focused on workload ~~experienced by~~of nurses ~~and therefore~~; other professionals such as physicians, physician-assistants, and paramedics were not included in this study.

Factors that consider employees other than nurses (such as nurse-physician relationships, support from logistic teams), factors that cannot be influenced by nurse management of the ward (such as social support at home) or factors that require major investments (such as ward layout and number of single rooms in a ward) were not included in the scope.

Design

In this ~~paper~~research, five different measures of perceived workload are included: work pace, amount of work, mental workload, emotional workload and physical workload. The job demands (interruptions, work distribution, time for registration, time for direct patient care) and job resources (support from colleagues, support from management, the proportion of registered nurses on the ward) are included in the analyses as potential effect modifiers of the relation between modelled and perceived workload. Effects will be explored separately for each outcome measure. presents a visualization of the model to be studied.

Formatted: Font: Bold

Formatted: Font: Bold

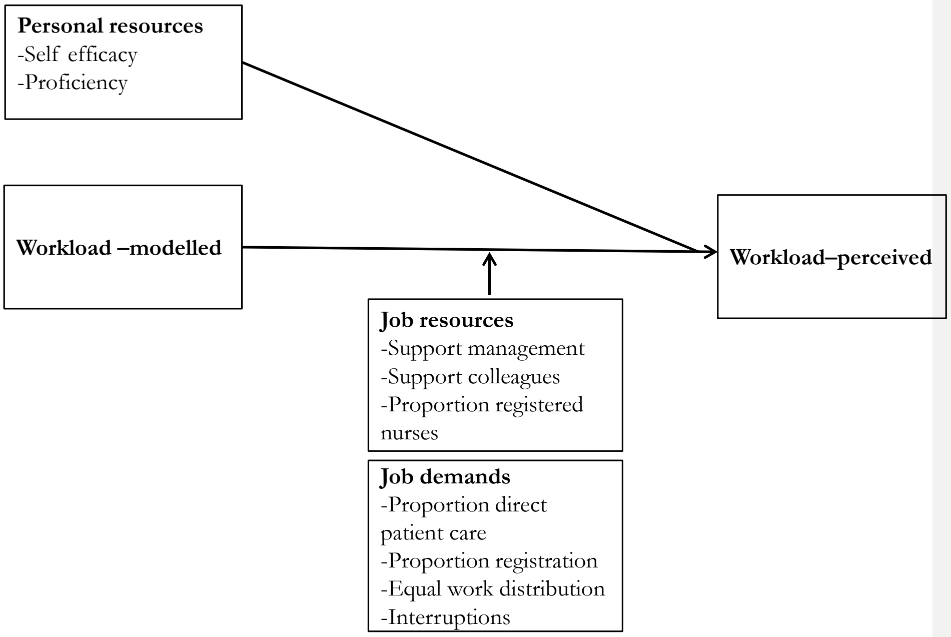

Figure 1: Studying the relation between modelled and perceived workload (our hypotheses)

Data collection

Data was collected in four ways:

1. An observational work sampling study of the activities of nurses was done on fifteen consecutive day shifts. Details on the study can be found in previous publication of the study team [41]. This study yielded estimates of time spent on direct patient care, collective patient care, general tasks and other tasks.
2. During the observational study period, previously selected relevant patient characteristics ~~[39]~~were registered for all patients admitted on the study wards[40]. After the work sampling study, a linear mixed effects model was used to determine significance and care time estimates for each characteristic [~~39,~~ 40]
3. On each day of the observational study, each nurse on duty during the dayshift was asked to fill out a short questionnaire. The questionnaire was filled out at the end of the shift and contained questions on job demands, job resources, engagement, quality of care and stress

Formatted: Font: Bold

Formatted: List Paragraph, Numbered + Level: 1 + Numbering Style: 1, 2, 3, … + Start at: 1 + Alignment: Left + Aligned at: 0.25" + Indent at: 0.5"

4. Three weeks prior to the observational study, all nurses employed (as registered nurses or students) on the study wards were asked to fill out an extensive questionnaire on job demands, job resources, personal resources and personal and job characteristics.

Formatted: Font: (Default) Garamond, 12 pt

Measures

Formatted: Font: Bold

Outcome measures: perceived workload

Formatted: Font: Italic

Perceived workload was measured with items derived from the Questionnaire on the Experience and Evaluation of Work (QEEW), which is widely used by Dutch occupational health services to measure the (psychosocial) working environment [44]. The five measures for perceived workload were:

'Did you have too much work to do today', reflecting the amount of work;

'Did you have to work very fast today', reflecting work pace;

'Did you consider your work mentally very challenging today', reflecting mental workload;

'Did your work demand a lot from you emotionally today', reflecting emotional workload;

'Did you find your work physically strenuous today', reflecting physical workload.

Each item had a five-point response scale ranging from 'Not at all' (1) to 'Very often' (5). All measurements were done during the work sampling study period, on a daily basis at the end of the shift.

Independent variable: modelled workload

Formatted: Font: Italic

As mentioned, previous work of this study team contained a study design for developing a workload management method [38]. This method consisted of 7 steps. In step 1, a group of expert nurses composed a list of fifteen patient characteristics they expected to be most relevant to care time. Subsequently, an observational study on nurses' activities (step 2) measured how much time nurses spent on direct patient care ~~(care attributed to an individual patient)~~, collective patient care ~~(patient-related tasks but not attributable to individual patients, e.g. meal distribution)~~ , general tasks ~~(other activities that were not directly patient related such as general administration, meetings, education)~~ and other tasks ~~(breaks and personal time)~~ [41]. The relation between the patient characteristics and care time was studied in step 3 ~~*(manuscript accepted and in preparation for publication in Nursing Open)*~~[40], n~~. N~~ine of the fifteen patient characteristics ~~were~~ turned out to be significantly related to care time~~: 'partial assistance bathing/ mobilization', 'full assistance bathing/ mobilization', 'full assistance meals/nutrition', 'two or more IV/drip/drain', 'psychosocial support', 'extensive wound care', 'stoma care', 'isolation measures', 'one on one care'~~. In addition to care time related to patient characteristics, it was also assumed that there

would be a patient-related 'baseline care time'. When a patient is admitted to a ward, nurses spend a certain amount of care time on this patient e.g., time that is spent on handing out meals, having a chat or tidying up) , regardless of the patient characteristics that apply. The observational study also yielded how much time nurses spent on non-patient related activities (step 4).The objective modelled workload (step 7) is then calculated by comparing required nursing time (step 5) to allocated nursing time (step 6). The total required nursing time is the result of adding up care time related to patient characteristics, baseline patient related care time and time for non-patient related activities. These times were derived from a time study [41], in which all nurses of the six departments in the study were observed during the day shift, over a period of fifteen working days. During the time study period, nurses on duty registered relevant patient characteristics for each patient each day shift.

Total required nursing time was calculated as follows:

$$(a1 * n1 + a2 * n2 + a.. * n.. + ..) + (b * N) = x$$

$$Yreq = \frac{x}{(1 - z)}$$

$a1 = additional\ care\ time\ when\ patient\ characteristic\ 1\ applies\ (minutes)$

$n1 = number\ of\ patients\ in\ the\ dayshift\ for\ whom\ characteristic\ 1\ applies$

$b = baseline\ care\ time\ per\ patient\ (minutes)$

$N = number\ of\ patients\ admitted\ in\ the\ dayshift$

$x = amount\ of\ care\ time\ for\ patient\ related\ activities\ (minutes)$

$z = \%\ of\ time\ spent\ by\ nurses\ on\ non - patient\ related\ activities$

$\boldsymbol{Yreq = total\ required\ nursing\ time\ (minutes)}$

Both required and allocated nursing times were calculated with corrections for nurse proficiency. In the work sampling source data, care times were corrected directly for nurse proficiency[40]. These corrected care times were the basis for calculating the require nursing time.

Allocated nursing time was determined by adding up the amount of nurses in the shift, multiplying this by the shift time and correcting for nurse proficiency. Proficiency was estimated by expert nurses. In a mini Delphi study, all six ward managers (all nurses with more than fifteen years of experience) of the wards involved in the study were asked to define nurse categories and corresponding proficiency levels. The mini Delphi consisted of two rounds and two sessions to discuss results. The Delphi yielded six nurse proficiency categories. One The reference category is the fully qualified and experienced registered nurse: this was defined as the standard and set to a proficiency percentage of 100%. The proficiencies of the other five types of nurses (novice

registered nurses, fulltime student nurses in their first or second year, fulltime student nurses in their third or fourth year, working student nurses in their first or second year, working student nurses in their third or fourth year) were offset against this standard.
The allocated nursing time is calculated as follows:

$$(m1 * p1 + m2 * p2 + m.. * p.. + ..) = Yall$$

$m1 = amount\ of\ time\ nurse\ 1\ was\ observed\ in\ the\ day\ shift\ (minutes)$

$p1 = proficiency\ of\ nurse\ 1$

$\boldsymbol{Yall = total\ allocated\ nursing\ time\ (minutes)}$

The modelled workload was calculated by dividing required nursing time by allocated nursing time. This ratio gives an objective indication of nurses' workload (W). W = 100% reflects that the allocated nursing time is perfectly balanced to the required nursing time; W<100% indicates overstaffing and W>100% understaffing.

$$W = 100\% * \frac{Yreq}{Yall}$$

$\boldsymbol{Yreq = total\ required\ nursing\ time\ (minutes)}$

$\boldsymbol{Yall = total\ allocated\ nursing\ time\ (minutes)}$

$\boldsymbol{W = modelled\ workload\ (\%)}$

This modelled workload was calculated retrospectively for each ward on each of the day shifts during the time study period.

Covariates: personal resources, job resources and job demands

Formatted: Font: Italic

Several covariates are included in the analysis, in order to discover potential interventions for balancing perceived workload in daily practice.
~~Data on p~~Personal resources ~~in our study~~ were not expected to vary on a daily basis and were measured once at baseline, three weeks prior to the work sampling period. Personal resource proficiency was estimated by expert nurses, as mentioned above. Personal resource self-efficacy was measured by a scale ~~widely used by Dutch occupational health services in the context of occupational health surveillance. The scales are~~ based on Schwarzer & Jerusalem's general self-efficacy scale [65]. The scale contains five questions: 'When difficult problems occur at work, I know how to deal with them', 'At work, I reach my goal, even when unexpected situations occur', 'If I encounter obstacles at work, I always find a way around them', 'Even if it takes a lot of my

time and energy, at work I achieve what I want' and 'If I encounter something new at work, I always know how to deal with it'. All questions had a five point answer scale, ranging from 'Agree completely' (5) to 'Disagree completely' (1). Scores on the five questions were summed to a total score and then averaged per respondent.

Job resources support from management and support from colleagues were measured on a daily basis for all nurses on duty during the time study period. The questions originated from the Questionnaire on the Experience and Evaluation of Work (QEEW[35]). Support from management was measured by the question 'I could count on my supervisor when I came across difficulties in my work today'. Support from colleagues was determined by the question 'I could count on my colleagues when I came across difficulties in my work today'. Both questions had a 5 point answer scale, ranging from 'Not at all' (1) to 'Very often' (5).

The job resource percentage of registered nurses on the ward was determined by mapping the nurse qualifications of all nurses involved in the study and calculating a percentage of registered nurses on duty on each ward for each day of the time study.

The job demands perceived equality of work distribution and perceived interruptions were also measured once at baseline. Perceived equality of work distribution was measured by the question 'Is the work distributed evenly across all employees of the department?'. Perceived interruptions was determined by the question "Do you have to deal with interruptions in your work?". Both questions had a four point answer scale, ranging from 'Never' (1) to 'Always (4). These questions were ~~also~~ derived from ~~the~~ questionnaires often used by Dutch occupational health services.

The proportions of time spent on direct patient care and time spent on registration were derived from the ~~time~~ observational study results [41] and determined per nurse per day. Direct patient care entails all activities that can be attributed to one specific patient, excluding registration. Registration is defined as all administrative and registration tasks done either on behalf of the patient (such as patient documentation, ordering medication and applying for tests and examinations) or as part of general administrative activities (such as ordering supplies, processing incoming mail).

~~Statistical~~ Data analysis

A one-way ANOVA and Tukey post-hoc tests were performed to determine significant differences between wards for the modelled workload.

Linear mixed effects models [66] were used to study the relation between the modelled workload and perceived workload. Observations were not independent since the majority of nurses were observed more than once during the work sampling period (longitudinal data). Linear mixed

effects models are suitable for analyzing longitudinal data. The modelled workload *W* was the independent variable and the five perceived workload measures were the outcome variables; each model included a random intercept per nurse to account for dependence of measures within nurses over time.

For each of the five perceived workload measures, the relation between the modelled workload *W* and the perceived workload was first tested, with the wards as fixed effects. Subsequently, the personal resources (2 variables), job resources (3 variables) and job demands (4 variables) were added to the model in blocks to test the direct effects on perceived workload. In the last step, interactions between the modelled workload estimate and the four job demands and three job resources were introduced, to test for moderation. This resulted in five estimated models for each of the five outcome measures.

The significance level was set to 0.01. Model fits were evaluated for each outcome measure by comparing the Bayesian Information Criterion (BIC) values. BIC was chosen over the Akaike information criterion and likelihood ratio testing because the models include a relatively large number of independent variables and BIC is more conservative when testing several parameters at once.

Ethical considerations

The study guaranteed the privacy of involved staff and patients. The study protocol was reviewed and approved by the medical ethical review board of the UMC Utrecht, protocol number 14-165/C.

Results

Baseline and daily questionnaires

The daily questionnaire was filled out 694 times, resulting in an average response rate of 58%. The baseline measure questionnaire was returned by 162 nurses; a response rate of 65%. Details on response rates, population characteristics and average responses for personal resources and job resources are shown per ward in **Table 1**.

Formatted: Left: 0.98", Right: 0.98", Top: 0.98", Bottom: 0.98", Section start: Continuous, Header distance from edge: 0.49", Footer distance from edge: 0.49", Numbering: Continuous

Table 1: Response rates, population characteristics and average response of the respondents of the baseline and daily questionnaires

	Ward	1				2				3				4				5				6			
**Response**		n	%	Mean	SD	n	%	Mean	SD	n	%	Mean	SD	n	%	Mean	SD	n	%	Mean	SD	n	%	Mean	SD
**Baseline response**		**28**	**72%**			**33**	**73%**			**29**	**54%**			**31**	**62%**			**16**	**53%**			**25**	**83%**		
**Gender**	Male	2	7%			3	9%			5	17%			6	19%			0	0%			2	8%		
	Female	26	93%			30	91%			24	83%			25	81%			16	100%			23	92%		
**Age category**	<20	1	4%			0	0%			0	0%			0	0%			0	0%			1	4%		
	20-30	10	36%			15	45%			17	59%			21	68%			8	50%			13	52%		
	30-40	3	11%			9	27%			4	14%			3	10%			2	13%			3	12%		
	40-50	3	11%			4	12%			1	3%			1	3%			0	0%			2	8%		
	>=50	8	29%			2	6%			3	10%			2	6%			5	31%			5	20%		
	Unknown	3	11%			3	9%			4	14%			4	13%			1	6%			1	4%		
**Type of nurse**	Registered nurse	24	64%			31	70%			20	52%			21	58%			10	44%			16	48%		
	Student nurse	4	36%			2	30%			9	48%			10	42%			6	56%			9	52%		
**Self-efficacy (range 1-5)**				3,5	0,4			3,6	0,5			3,6	0,4			3,7	0,4			3,6	0,4			3,6	0,5
**Interruptions (range 1-4)**				2,5	0,8			2,7	0,7			2,3	0,7			2,3	0,8			2,4	0,8			2,0	0,9
**Equal work distribution (range 1-4)**				2,8	0,5			2,5	0,7			2,7	0,6			2,6	0,7			2,8	0,4			2,6	0,7
**Daily response**		**91**	**58%**			**107**	**45%**			**141**	**63%**			**151**	**66%**			**93**	**57%**			**111**	**61%**		
**Gender**	Male	15	16%			3	3%			36	26%			30	20%			8	9%			12	11%		
	Female	68	75%			96	90%			102	72%			95	63%			80	86%			91	82%		
	Unknown	8	9%			8	7%			3	2%			26	17%			5	5%			8	7%		
**Age category**	<20	3	3%			0	0%			0	0%			11	7%			4	4%			12	11%		
	20-30	49	54%			66	62%			106	75%			98	65%			62	67%			67	60%		
	30-40	8	9%			16	15%			12	9%			9	6%			19	20%			18	16%		
	40-50	13	14%			15	14%			8	6%			4	3%			0	0%			3	3%		
	>=50	10	11%			2	2%			12	9%			3	2%			3	3%			3	3%		
	Unknown	8	9%			8	7%			3	2%			26	17%			5	5%			8	7%		
**Type of nurse**	Registered nurse	62	68%			70	65%			68	48%			85	56%			51	55%			41	37%		
	Student nurse	29	32%			37	35%			73	52%			66	44%			42	45%			70	63%		
**Support management (range 1-5)**				3,8	1,1			3,7	1,2			4,1	1,1			3,9	1,0			3,9	1,1			4,1	1,0
**Support colleagues (range 1-5)**				4,1	0,8			4,0	0,9			4,2	0,9			4,0	0,9			4,2	0,8			4,3	0,8

Not surprisingly, the vast majority of nurses are female (**Table 1**). Of the respondents of the baseline questionnaire, between 44% and 70 % were registered nurses; in the daily questionnaires this ranged between 37% and 68%. Gender and age category could not always be ascertained, for example for students who left the hospital or for temporary nursing staff. 36% to 68% of respondents were between 20 and 30 years old. The relatively high proportion of young nurses can be explained by the study setting: the research took place in an academic hospital, with specific training and educational tasks. On all wards, support of colleagues scored higher on average than support of management (4.0 to 4.3 and 3.7 to 4.1, respectively). Self-efficacy seems quite stable across wards, with average scores ranging between 3.5 and 3.7 and standard deviations between 0.4 and 0.5.

Modelled workload

One of the main practical concerns in the study hospital was whether the workload was divided equally across wards. In order to answer this question, modelled workload was analyzed per ward. **Figure 2** presents the average modelled workload per day shift, calculated retrospectively for the days of the work sampling period. Each line represents one ward. During the work sampling period, on two day shifts equipment failed on a ward and on one day the data download failed for one ward. This meant that not all the required data were available to calculate the modelled workload on those days, so three day shifts were excluded for all wards. Days 1, 3 and 5 are missing. Since these failures occurred randomly, it is expected that the missing data do not influence the outcomes.

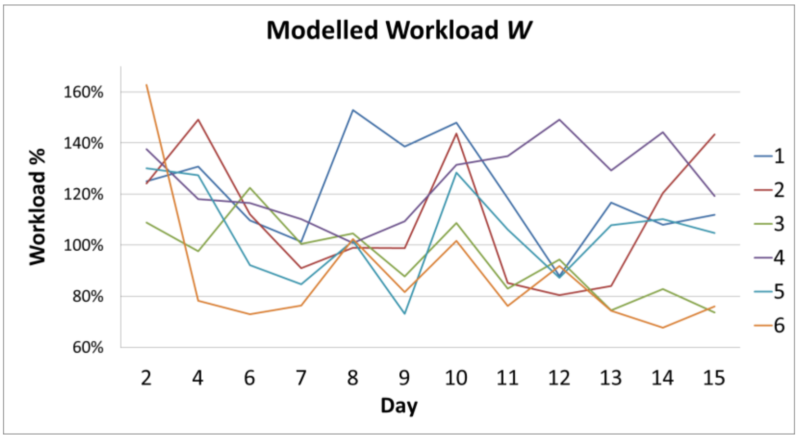

Figure 2: Average modelled workload per ward per day, calculated retrospectively for the work sampling period

The post hoc tests found that modelled workload on ward 6 was significantly lower than on wards 1 (estimated difference -32.3%, confidence interval -58.1% to -6.4%, p-value 0,001) and 4 (estimated difference -36.6%, confidence interval -62.4% to -10.7%, p-value <0,001), and workload on ward 3 was significantly lower than on ward 4 (estimated difference 30.2%, confidence interval -56.0% to -4.3%, p-value 0,002).

Perceived workload

For the perceived workload measures, data were available for all fifteen day shifts in the work sampling period.

Figure 3 presents the average perceived workload per ward per day shift, for each of the five perceived workload measures.

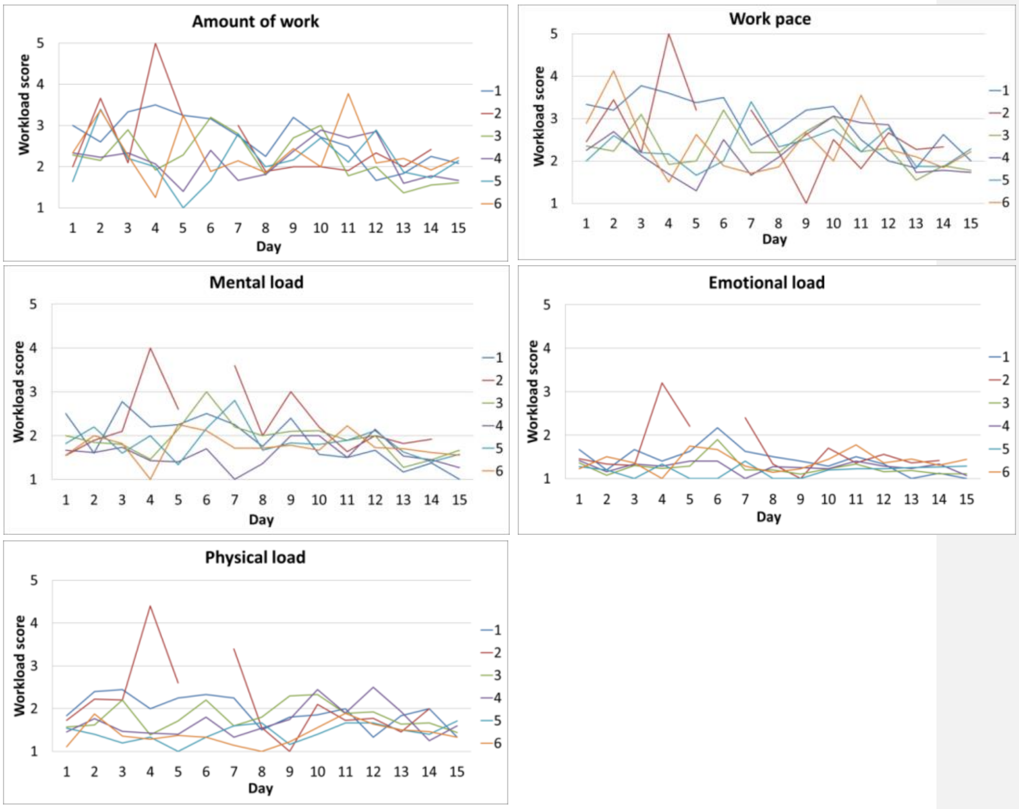

Figure 3: Five line graphs with average perceived workloads per ward per day for amount of work, work pace, mental load, emotional load and physical load

The graphs of amount of work and work pace are quite similar. The graph of emotional load is more stable and on average lower than the other measures. To a lesser extent, the same goes for the results on physical load. On day 4 on ward 2, workload was apparently perceived exceptionally high on all of the five outcomes measures. On day 9 on ward 2, all workloads were perceived to be relatively low, but mental load peaked.

Effects of personal resources, job resources and job demands

For all five perceived workload measures, the models with interactions did not perform better than the models without interactions, hence the interactions are not shown in Table 2.. This means that no moderation was found on the relation between modelled and perceived workload, all significant effects of variables were direct effects on perceived workload. Details on model fits can be found in **Appendix 1**.

For three outcome measures (mental load, emotional load and physical load), the models that included the job demands did not perform better than the models without the job demands. Therefore, no results are shown for the job demands for these three outcome measures.

Table 2: Effects of job demands, job resources and personal resources on five outcome measures of perceived workload (range 1 to 5)

		**Perceived Work pace**		**Perceived Amount of work**		**Perceived Mental load**		**Perceived Emotional load**		**Perceived Physical load**	
**Type of variable**	**Variable**	Estimated effect *	Standard error	Estimated effect *	Standard error	Estimated effect *	Standard error	Estimated effect *	Standard error	Estimated effect *	Standard error
**Job demand**	Modelled workload	**0.209**	**0.024**	**0.198**	**0.025**	**0.141**	**0.021**	**0.043**	**0.013**	**0.047**	**0.017**
**Personal resource**	Perceived self-efficacy	0.010	0.211	-0.03	0.248	-0.12	0.212	-0.08	0.147	-0.10	0.218
	Estimated nurse proficiency %	-0.00	0.039	-0.03	0.046	0.008	0.037	-0.00	0.026	-0.00	0.038
**Job resource**	Perceived support of management	-0.01	0.057	-0.05	0.061	-0.08	0.052	-0.02	0.032	-0.03	0.043
	Perceived support of colleagues	**-0.23**	**0.073**	**-0.30**	**0.079**	**-0.23**	**0.067**	**-0.11**	**0.042**	**-0.22**	**0.057**
	% of nurses on ward that is registered nurse	0.088	0.057	0.068	0.061	0.083	0.052	**0.085**	**0.032**	0.076	0.044
**Job demand**	% nursing time spent on direct patient care	**0.165**	**0.037**	**0.228**	**0.040**	.	.	.	.	.	.
	% nursing time spent on registration	-0.19	0.080	**-0.29**	**0.085**	.	.	.	.	.	.
	Perceived equality in work distribution	-0.10	0.171	-0.15	0.201	.	.	.	.	.	.
	Perceived interruptions	0.232	0.120	0.150	0.142	.	.	.	.	.	.

*For variables with ordinal scales (for example from "Never" to "Always", translated into 1 to 4 points), a rise of one full point results in the effect shown in Table 2. For ratio variables, a rise of 10% results in the effect shown in Table 2.
Significant effects are printed in bold.

The results indicate a positive correlation between all perceived workload measures and modelled workload. Every 10% increase in estimated workload (on a scale that ranges roughly between 70 and 170%) is associated with a 0.209 increase in perceived work pace (on a scale from 1 to 5 this is a 5.2% increase), 0.198 (5%) in perceived amount of work, 0.141 (3.5%) in perceived mental workload, 0.043 (1.1%) in perceived emotional workload and 0.047 (1.2%) in perceived physical load.

Neither of the personal resources were significantly related to any of the perceived workload measures. The scale for self-efficacy was tested for internal consistency by calculating Cronbach's alpha. Alpha was 0.657 and did not improve if items were deleted from the scale. Since this practical scale is used often by occupational health services, it was decided to keep the scale in the study and test whether it was of influence on perceived workload even though Cronbach's alpha was relatively low.

Support from management was not related to any of the perceived workload measures, whereas support from colleagues was negatively associated with all outcome measures. A 1-point increase (on an scale of 1 to 5) in experienced support from colleagues reduced the experienced work pace with 0.23 (5.8%), amount of work with 0.30 (7.5%), mental workload with 0.23 (5.8%), emotional workload with 0.11 (2.8%) and physical load with 0.22 (5.5%).

The percentage of registered nurses on the ward is correlated with the perceived emotional workload, but not the other subjective workload outcomes. Every 10% increase in proportion of registered nurses on a ward results in 0.085 (2.1%) increase in experienced emotional workload. The proportion of time spent on direct patient care and the proportion of time spent on registration were significantly related to perceived workload. For each 10% increase in time spent on direct patient care, there was a 0.165 (4.1%) point rise in perceived work pace and 0.228 (5.7%) point rise in perceived amount of work. Every 10% increase in proportion of time spent on registration gave a 0.29 (7.3%) decline in perceived amount of work. The other job demands were not significantly related to any of perceived workload measures.

Proportional effects for significant variables are summarized in **Table 3**.

Table 3: Proportional effects for variables with significant effect on perceived workload

			**Perceived Work pace**	**Perceived Amount of work**	**Perceived Mental load**	**Perceived Emotional load**	**Perceived Physical load**
**Type of variable**	**Variable**	**Increase**	Estimated proportional effect*	Estimated proportional effect*	Estimated proportional effect*	Estimated proportional effect*	Estimated proportional effect*
**Job demand**	Modelled workload	+10%	+5.2 %	+5 %	+3.5 %	+1.1 %	+1.2 %
**Job resource**	Perceived support of colleagues	+1 point	-5.8 %	-7.5 %	-5.8 %	-2.8 %	-5.5 %
	% of nurses on ward that is registered	+10%				+2.1 %	
**Job demand**	% nursing time spent on direct patient care	+10%	+4.1 %	+5.7 %			
	% nursing time spent on registration	+10%		-7.3 %			

*Estimated proportional effect on the outcome variable (expressed in % of rise in points on a 1-5 scale) for a rise of either 10% or 1 point in the independent variable

Discussion

Findings

The modelled workload is significantly different between wards 6 and 1, between 6 and 4 and between 3 and 4. The estimated differences are quite large; -32.3%, -36.6% and -30.2% respectively but so are the corresponding confidence intervals: -58.1% to -6.4%, -62.4% to -10.7% and -56.0% to -4.3% respectively.

The first hypothesis was accepted, since there is a linear correlation between the modelled workload and all five perceived outcome measures. Every 10% increase in modelled workload results in a 0.209 (5.2%) increase in perceived work pace, 0.198 (5%) in perceived amount of work, 0.141 (3.5%) in perceived mental workload but only 0.043 (1.1%) in perceived emotional workload and 0.047 (1.2%) in perceived physical load. Apparently, modelled workload W has the biggest effect on work pace, amount of work and mental workload. This makes sense, considering that the modelled workload W is based on comparing required and allocated care time only, and does not give any insight in emotional and physical requirements. Having to work fast due to a lack of balance between required and allocated resources may result in an experienced higher mental load because tasks that need focus and concentration need to be done under time pressure. Modelled workload, as found in this study, varies roughly between 70% and 170%; a range of 100%. With every 10% increase of the modelled workload, the perceived work pace increases with 5.2% and the perceived amount of work with 5.0%. It seems that perceived workload does not rise to the same extent as modelled workload. However, we need to consider that the answer range that was provided for all perceived workload measures ranges from 1 to 5 on an ordinal scale, which is quite a narrow range. Respondents therefore had limited options to express their perceptions of workload. In future research, a broader range of response may be of value to get a more detailed insight on the extent to which modelled workload influences perceptions.

Of the perceived workload measures, work pace and amount of work show a similar pattern. In the QEEW [35] these two are brought together in one construct: work pressure. In our study, we made the decision to separate the two in order to see whether the covariates may have different effects on each measure. Indeed this turned out to be the case; percentage of time spent on registration was correlated with perceived amount of work but not with work pace. Apparently, researching these two measures as separate constructs pays off. The graph of emotional load is most stable and on average reports a lower workload than the other measures. To a lesser extent, the same goes for the results on physical load. There were two peak days for ward 2 (days 4 and 9), we could not retrieve what would have been the reason for this. Especially the peak in mental

workload on day 9 is puzzling, since all other workloads measures on that ward on that day were relatively low.

Personal resources self-efficacy and proficiency were not related to any of the outcome measures. As for self-efficacy, this is not in line with findings in other studies, such as those of Spence Laschinger [60] and Martens [62], where positive correlations were found between (occupational coping) self-efficacy and workload and stress of staff shortages. Our findings do correspond with Schmidt's[51], who did find an effect of self-efficacy on job strain but not on perceived workload. However, the internal consistency of the scales for self-efficacy used in our study turned out to be low. The questions in the scale are insufficiently correlated to be seen as one construct, so no definite conclusion can be drawn about the effect of self-efficacy on perceived workload. It is recommended that in further research, other validated scales for self-efficacy are used. A study on nurse risk assessment decisions [67], showed that under conditions without time pressure, nurses with clinical expertise (i.e. more proficient nurses) performed better than novice nurses; the positive effects of clinical expertise, however, were negated when time pressure was introduced to clinical simulations. Since proficiency of nurses was not significantly related to workload in our study and in addition the workload estimate W was higher than 100% in 4 out of 6 wards, which indicates understaffing, possibly, this effect also occurred in our study setting.

There was no evidence that the personal resources are related to the perceived workload measures; no direct effects or moderation were found, so hypothesis two was rejected.

Of the job demands other than workload, only the proportions of direct patient care and registration were of significance. An increased proportion of direct patient care results in increased perceptions of work pace and amount of work. This may be explained by the fact that time spent with the patient is regarded as more demanding; possibly patient related tasks are considered more urgent than other tasks and add to perceived work pressures. In line with this finding, an increase in proportion of time spent on registration gave a decline in perceived amount of work. Registration might be regarded as a less demanding task which may influence the perception of the work pressure. More research is needed on this, but the results may be explained by the fact that registration takes place in a quiet environment, away from the patient and thus away from potential pressures of patients and relatives or other caregivers. Possibly administrative work can form a stable, quiet moment in the working day where nurses can focus on this one task, whilst sitting down at a desk, instead of having to hurry along between tasks in different patient rooms. Also, unexpectedly, perceived work interruptions seemed irrelevant to perceived workload. Based on the available literature [46, 48], which shows a negative correlation between interruptions and patient safety, for example when performing complicated tasks such as

medication preparation, a positive correlation between interruptions and perceived workload was expected, at least with outcome measure perceived mental load. Perceived equality in work distribution was also not proven to be correlated to any of the workload outcome measures. To our knowledge there is no available literature on the effects of equal work distribution in the day shift on perceived workload. There are studies on distributive organizational justice (which reflects the perceived fairness in decision outcomes, such as work scheduling), and these suggest a negative correlation between distributive justice and intention to leave [68] and a positive correlation between distributive justice and quality of work life (for example psychological well-being, workload and work satisfaction)[69]. Assuming that equal work distribution would be also perceived as a fair distribution, a positive correlation between equal work distribution and perceived workload was expected but not proven. However, upon studying the different model fits for all outcome measures, there was a notable finding. For outcome measure perceived mental workload, two models scored almost similar on the BIC (see **Appendix 1**, **Table 3**): the model with all personal and job resources and all job demands (BIC=1061.9) and the model that also included interactions (BIC=1062.1). The model with interactions showed a significant moderation on the relation between modelled and perceived workload, for job demands work interruptions and equality of work distribution.

The job resources support from colleagues and proportion of registered nurses were significantly related to perceived workload. Support from colleagues proved to be an important factor in workload perception, since it was negatively correlated to all outcome measures. This is in line with findings of other studies [7, 53, 54], where teamwork was shown to offset negative effects of high workload. The fact that the effect of teamwork was found to be less strong on outcome measure perceived emotional workload was unexpected; apparently support from colleagues is more important in handling workload in the cognitive and physical domain than when coping with emotional challenges. The percentage of registered nurses on the ward is positively correlated with the perceived emotional workload, although not very strong. It was expected that registered nurses would be better equipped to handle emotional stressors than student nurses, but apparently, there is more to this. Possibly, registered nurses feel more responsible for their work than student nurses, which may increase emotional load. Or maybe registered nurses are more likely to be given the more emotionally challenging tasks than student nurses. More research is needed to explain this effect. Perceived support from management did not turn out to be significantly related to any of the outcome measures. A study by MacPhee [14] did show that support of unit-level management in managing workload could have positive effects on perceived quality of care and job outcomes, but this study did not test the direct relation between support

of management and perceived workload. In the present study, the relation between support from management and workload was tested, but could not be proven. When considering which support is more important to nurses, apparently sticking together as a team is more important to how workload is perceived than getting support from ward leadership. This suggests that ward leadership should consider their role in enhancing teamwork and teambuilding on the ward.

For all outcome measures, the models without the interactions showed a better fit than the models with interactions. This means that there is only evidence for direct effects, there is no moderation, which means that hypotheses three and four must be rejected. More research is recommended on the effects of perceived interruptions and perceived equality of work distribution on perceived mental workload, because the model that included moderation was almost as good as the model without.

Strengths and limitations

This was a longitudinal, multilevel study, which is rare in this field of research. The fact that an objective (modelled) and subjective (perceived) workload measures were combined in one study is unique.

Another strong feature of this study was that nurses were in the lead and had complete freedom in determining a set of patient characteristics that they believed influence care time the most. This resulted in a new set of characteristics, several of which have not been studied before, such as a patient's bodily proportions and one-on-one care. The effects of the patient characteristics on care time were quantified by work sampling over a relatively long study period of 15 day shifts. Nurses involved in the study stressed the importance of nurse proficiency in workload calculations and suggested a new way of defining nurse proficiency. Data were corrected for this newly defined nurse proficiency, which has not been done before and proved to be an important factor to include in the analysis. The proportion of students is relatively high in our study, since the study setting is an academic hospital and two of the wards in the study are also specific learning wards which have a higher proportion of students than usual. Correcting for nurse proficiency is especially relevant in a teaching hospital setting.

A new concept 'baseline care time' was defined and quantified by deriving estimated marginal means from the mixed model, which has not been described in literature before.

The combination of a modelled workload measure and five perceived workload measures in one study is unique. Also, testing for moderation of job demands and job resources on the relation between the modelled and perceived workloads has not been done before.

The overall response rates on the baseline measure questionnaire and the daily questionnaire that

was used during the work sampling period were 65% and 70% respectively, which is considered to be quite good [70].

There are several limitations that need to be addressed. This study was set in an academic hospital, which makes it uncertain whether the study results can be readily applied to different settings, such as general hospitals. Nurses' activities and the patient mix in general hospitals are likely to be different than in academic hospitals, if this study is to be applied in such a setting, the framework can be the same, but it is recommended to review the list of patient characteristics and to repeat the work sampling. The same goes for departments with a unique nature, such as pediatric and psychiatric wards.

The confidence intervals for the estimated differences of modelled workload between wards were relatively high. For future research, a larger sample size is recommended in order to make a more accurate estimate of differences between wards.

The scales used to measure self-efficacy were chosen for practical reasons and were not validated by means of international publications. The items on the scale proved not to be internally consistent; future research should include validated scales of self-efficacy. Since the items do not represent one construct, no conclusion can be drawn about the effect of self-efficacy on perceived workload.

Perceived equality of work distribution and perceived interruptions were both measured by one question. Possibly, the use of more extensive measures would have influenced results. More research is recommended on this.

In this study, proficiency was estimated by a mini-Delphi study among head nurses. Another way to measure the proficiency of nurses would be to keep track of the actual exact time spent on each activity, calculate an estimate per activity per type of nurse and derive the proficiency percentage from these estimates. However, since there were 6 types of nurses and 24 activity groups in the study, this approach would have required a much larger sample size and a more accurate measurement of time spent on an activity than work sampling every ten minutes. For practical reasons (costs, registration), this was not possible, and the choice was made to estimate nurse proficiency instead.

Lastly, the work sampling study was done during fifteen consecutive dayshifts, which is quite a long time period but also only one time period. Possibly, results would have been more powerful if the study was done over two separate time periods. However, due to practical issues we did not chose this option. If the study was set over two time periods, staff would need to be trained and informed twice (for example not all medical students would be available for both time periods

and new nursing students would have entered the wards), equipment would need to be rented twice and the preparations such as barcoding patient rooms would need to be done again. This would have become too costly so the study team opted against this.

Concluding remarks

The study presents a method to calculate an objective measure for workload of nurses, which is positively associated with the perceived workload. This modelled workload can be used to detect differences in workload between wards, which may be useful to more evenly distribute workload in order prevent issues in the domain of e.g. organizational justice. When sufficient historical data are collected, an average patient characteristics profile per admission day can be composed for treatments such as operations. This way, an indication of workload of nurses on surgical hospital wards can be predicted based on operating room schedules. Besides the modelled workload, job resources support of colleagues and job demands time spent on direct patient care and time spent on registration had the biggest significant effects on perceived workload. In times when workload is high, extra effort in teambuilding is likely to have a positive effect on perceived workload. In these circumstances, unit-level management should consider focusing their attention on facilitating the nursing team to work together smoothly and on enhancing team spirit. Since time spent on direct patient care is positively associated with perceived workload and time spent on registration is negatively associated with perceived workload, a good balance between time spent on direct patient care and registration may also benefit perceptions of workload.

The findings of this research can help nurse management in allocating resources and directing their attention to the most relevant factors so workload of nurses is better balanced, which in turn leads to a higher quality of care, keeping nurses healthy and the prevention of additional costs for overstaffing, absenteeism or high turnover of nurses.

References

1. Cuckler GA, Sisko AM, Poisal JA, Keehan SP, Smith SD, Madison AJ, Wolfe CJ, Hardesty JC: **National Health Expenditure Projections, 2017-26: Despite Uncertainty, Fundamentals Primarily Drive Spending Growth.** In: *Health Affairs.* vol. 37, 1 March 2018 edn; 2018.
2. Martín Martín JJ, Puerto López del Amo González M, Dolores Cano García M: **Review of the literature on the determinants of healthcare expenditure**. *Journal of applied economics* 2011, **43**(1):19-46.
3. Dall TM, Gallo PD, Chakrabarti R, West T, Semilla AP: **An Aging Population And Growing Disease Burden Will Require ALarge And Specialized Health Care Workforce By 2025**. *Health affairs* 2013, **32**(11).
4. Zhang X, Tai D, Pforsich H, Lin VW: **United States Registered Nurse Workforce Report Card and Shortage Forecast: A Revisit.** *American Journal of Medical Quality* 2017, **33**(3):229-236.
5. WorldHealthOrganization: **Global Strategy on Human Resources for Health: Workforce 2030**. In. Edited by Europe WHORof: World Health Organization; 2014.
6. European Commission: **Second Biennial Report on social services of general interest**. In. Edited by Directorate-General for Employment Social Affairs and Inclusion; 2011.
7. Van Bogaert P, Peremans L, Van Heusden D, Verspuy M, Kureckova V, Van de Cruys Z, Franck E: **Predictors of burnout, work engagement and nurse reported job outcomes and quality of care: a mixed method study**. *BMC Nurs* 2017, **16**:5.
8. Yu D, Ma Y, Sun Q, Lu G, Xu P: **A nursing care classification system for assessing workload and determining optimal nurse staffing in a teaching hospital in China: A pre-post intervention study**. *International journal of nursing practice* 2015, **21**(4):339-349.
9. Aiken LH: **Nursing staff and education and hospital mortality in nine European countries: a retrospective observational study**. *Lancet* 2014, **383**(9931):1824-1830.
10. Liu LF, Lee S, Chia PF, Chi SC, Yin YC: **Exploring the association between nurse workload and nurse-sensitive patient safety outcome indicators**. *The journal of nursing research : JNR* 2012, **20**(4):300-309.
11. Unruh L: **Nurse staffing and patient, nurse, and financial outcomes**. *The American journal of nursing* 2008, **108**(1):62-71; quiz 72.
12. Hinno S, Partanen P, Vehvilainen-Julkunen K: **Nursing activities, nurse staffing and adverse patient outcomes as perceived by hospital nurses**. *J Clin Nurs* 2012, **21**(11-12):1584-1593.
13. Källberg AS, Ehrenberg A, Florin J, Östergren J, Göransson KE: **Physicians' and nurses' perceptions of patient safety risks in the emergency department.** *International emergency nursing* 2017, **33**:14-19.
14. MacPhee M, Dahinten VS, Havaei F: **The Impact of Heavy Perceived Nurse Workloads on Patient and Nurse Outcomes**. *Administrative Sciences* 2017, **7**:1-17.
15. Van Bogaert P, Clarke S, Willems R, Mondelaers M: **Staff engagement as a target for managing work environments in psychiatric hospitals: implications for workforce stability and quality of care**. *J Clin Nurs* 2013, **22**(11-12):1717-1728.
16. Aiken LH, Sermeus W, Van den Heede K, Smith HL, Kutney-Lee A: **Patient safety, satisfaction, and quality of hospital care: cross sectional surveys of nurses and patients in 12 countries in Europe and the United States.** *BMJ* 2012, **344**(E1717).

17. Bakker A.B. DE, Verbeke W.: **Using the Job Demands-Resources model to predict burnout and performance**. *Human Resource Management* 2004, **43**:83-104.
18. Spence Laschinger HK, Grau AL, Finegan J, Wilk P: **Predictors of new graduate nurses' workspace well-being: testing the job demands-resources model**. *Health care management review* 2012, **37**(2):175-186.
19. Ohue T, Moriyama M, Nakaya T: **Examination of a cognitive model of stress, burnout, and intention to resign for Japanese nurses**. *Japan journal of nursing science : JJNS* 2011, **8**(1):76-86.
20. Toh SG, Ang E, Devi MK: **Systematic review on the relationship between the nursing shortage and job satisfaction, stress and burnout levels among nurses in oncology/haematology settings**. *International journal of evidence-based healthcare* 2012, **10**(2):126-141.
21. Mudaly P, Nkosi ZZ: **Factors influencing nurse absenteeism in a general hospital in Durban, South Africa**. *J Nurs Manag* 2015, **23**(5):623-631.
22. Van Bogaert P, van Heusden D, Timmermans O, Franck E: **Nurse work engagement impacts job outcome and nurse-assessed quality of care: model testing with nurse practice environment and nurse work characteristics as predictors**. *Frontiers in psychology* 2014, **5**:1261.
23. Lacey SR, Cox KS, Lorfing KC, Teasley SL, Carroll CA, Sexton K: **Nursing support, workload, and intent to stay in Magnet, Magnet-aspiring, and non-Magnet hospitals**. *The Journal of nursing administration* 2007, **37**(4):199-205.
24. Leone C, Bruyneel L, Anderson JE, Murrells T, Dussault G, Henriques de Jesus E, Sermeus W, Aiken L, Rafferty AM: **Work environment issues and intention-to-leave in Portuguese nurses: A cross-sectional study**. *Health Policy* 2015, **119**(12):1584-1592.
25. Twigg D, Duffield C: **A review of workload measures: a context for a new staffing methodology in Western Australia**. *Int J Nurs Stud* 2009, **46**(1):131-139.
26. Jones TL, Hamilton P, Murry N: **Unfinished nursing care, missed care, and implicitly rationed care: State of the science review.** *International journal of nursing studies* 2015, **52**:1121-1137.
27. Schaufeli WB, Bakker AB: **Job demands, job resources, and their relationship with burnout and engagement: a multi-sample study**. *Journal of organizational behaviour* 2004(25):293-315.
28. Schaufeli WB, Bakker AB, Van Rhenen W: **How changes in job demands and resources predict burnout, work engagement, and sickness absenteeism**. *Journal of organizational behavior* 2009, **30**:893–917.
29. Van Mol MMC, Nijkamp MD, Bakker J, Schaufeli WB, Kompanje EJO: **Counterbalancing work-related stress? Work engagement among intensive care professionals**. *Australian Critical Care* 2018, **31**:234-241.
30. Riedl E, Thomas J: **The moderating role of work pressure on the relationships between emotional demands and tension, exhaustion, and work engagement: an experience sampling study among nurses**. *European Journal of Work and Organizational Psychology* 2019, **28**(3):414-429.
31. Montgomery A, Spanu F, Baban A, Panagopoulou E: **Job demands, burnout, and engagement among nurses: A multi-level analysis of ORCAB data investigating the moderating effect of teamwork**. *Burn Res* 2015, **2**(2-3):71-79.

32. Myny D, Van Goubergen D, Gobert M, Vanderwee K, Van Hecke A, Defloor T: **Non-direct patient care factors influencing nursing workload: a review of the literature**. *J Adv Nurs* 2011, **67**(10):2109-2129.
33. Alghamdi MG: **Nursing workload: a concept analysis**. *Journal of nursing management* 2016, **24**(4):449-457.
34. Holden RJ, Scanlon MC, Patel NR, Kaushal R, Escoto KH, Brown RL, Alper SJ, Arnold JM, Shalaby TM, Murkowski K *et al*: **A human factors framework and study of the effect of nursing workload on patient safety and employee quality of working life**. *BMJ quality & safety* 2011, **20**(1):15-24.
35. Van Veldhoven M, Meijman TF: **Het meten van psychosociale arbeidsbelasting met een vragenlijst: de Vragenlijst Beleving en Beoordeling van de Arbeid [The measurement of psychosocial job demands with a questionnaire: the questionnaire on perception and evaluation of the work],**. In. Edited by Arbeidsomstandigheden NIv. Amsterdam; 1994.
36. Demerouti E, Bakker AB, Nachreiner F, Schaufeli WB: **The job demands-resources model of burnout**. *The Journal of applied psychology* 2001, **86**(3):499-512.
37. Bakker AB, Demerouti E: **Job demands-resources theory: Taking stock and looking forward**. *J Occup Health Psychol* 2017, **22**(3):273-285.
38. Oetelaar van den WFJM, Stel van HJ, Rhenen van W, Stellato RK, Grolman W: **Balancing nurses' workload in hospital wards: study protocol of developing a method to manage workload**. *BMJ Open* 2016, **6**(11).
39. Van den Oetelaar WFJM, van Stel HF, van Rhenen W, Grolman W: **Identifying patient characteristics related to care time: a Delphi study in surgical hospital wards**. *Submitted for Review* 2017.
40. Oetelaar van den WFJM, Rhenen van W, Stellato RK, Grolman W: **Balancing workload of nurses: Linear mixed effects modelling to estimate required nursing time on surgical wards**. *Nursing Open* 2019, **7**(1):235-245.
41. van den Oetelaar WFJM, Stel van HJ, Stellato RK, van Rhenen W, Grolman W: **Mapping nurses' activities in surgical hospital wards: a time study**. *PLOS ONE* 2017.
42. Rauhala A, Fagerström L: **Determining optimal nursing intensity: the RAFAELA method**. *Journal of advanced nursing* 2003, **45**(4):351-359.
43. Sermeus W, Delesie L, van den Heede K, Diya L, Lesaffre E: **Measuring the intensity of nursing care: making use of the Belgian Nursing Minimum Data Set**. *International journal of nursing studies* 2007, **45**(7):1011-1021.
44. Hoi SY, Ismail N, Ong LC, Kang J: **Determining nurse staffing needs: the workload intensity measurement system**. *J Nurs Manag* 2010, **18**(1):44-53.
45. Myny D, Van Hecke A, De Bacquer D, Verhaeghe S, Gobert M, Defloor T, Van Goubergen D: **Determining a set of measurable and relevant factors affecting nursing workload in the acute care hospital setting: a cross-sectional study**. *Int J Nurs Stud* 2012, **49**(4):427-436.
46. McGillis Hall L, Pedersen C, Fairley L: **Losing the Moment. Understanding Interruptions to Nurses' Work**. *The Journal of nursing administration* 2010, **40**(4):169-176.
47. Duffield C, Diers D, O'Brien-Pallas L, Aisbett C, Roche M, King M, Aisbett K: **Nursing staffing, nursing workload, the work environment and patient outcomes**. *Appl Nurs Res* 2011, **24**(4):244-255.

48. Redding D, A., Robinson S: **Interruptions and Geographic Challenges to Nurses' Cognitive Workload**. *Journal of nursing care quality* 2009, **24**(3):194–200.
49. Khademi M, Mohammadi E, Vanaki Z: **Resources–tasks imbalance: Experiences of nurses from factors influencing workload to increase**. *Iranian Journal of Nursing and Midwifery Research* 2015, **20**(4):476–483.
50. Bakker ABD, E; Euwema M.C.: **Job resources buffer the impact of job demands on burnout**. *Journal of Occupational Health Psychology* 2005(10):170-180.
51. Schmidt KH, Distel S: **Job demands and personal resources in their relations to indicators of job strain among nurses for older people**. *Journal of Advanced Nursing* 2012, **69**(10):2185–2195.
52. Bakker AB: **Job resources boost work engagement, particularly when job demands are high**. *Journal of Educational Psychology* 2007, **99**(2):274-284.
53. Yanchus NJ, Ohler L, Crowe E, Teclaw R, Osatuke K: **You just can't do it all': a secondary analysis of nurses' perceptions of teamwork, staffing and workload**. *Journal of Research in Nursing* 2017, **22**(4):313-325.
54. Sexton JB, Holzmueller CG, Pronovost PJ, Thomas EJ, McFerran S, Nunes J, Thompson DA, Knight AP, Penning DH, Fox HE: **Variation in caregiver perceptions of teamwork climate in labor and delivery units.** *Journal of perinatology* 2006, **26**(8):463-470.
55. Van Bogaert P, Kowalski C, Weeks SM, Van Heusden D, Clarke SP: **The relationship between nurse practice environment, nurse work characteristics, burnout and job outcome and quality of nursing care: a cross-sectional survey**. *Int J Nurs Stud* 2013, **50**(12):1667-1677.
56. Duffield C, Roche M, Merrick ET: **Methods of measuring nursing workload in Australia**. *Collegian* 2006, **13**(1):16-22.
57. Tourangeau A: **Nursing skill mix and experience reduce patient mortality**. *Hospital quarterly* 2002, **5**(3):19-20.
58. Morris R, MacNeela P, Scott A, Treacy P, Hyde A: **Reconsidering the conceptualization of nursing workload: literature review**. *J Adv Nurs* 2007, **57**(5):463-471.
59. Hegney D, Plank A, Parker V: **Nursing workloads: the results of a study of Queensland Nurses**. *Journal of nursing management* 2003, **11**(5):307-314.
60. Spence Laschinger HK, Borgogni L, Consiglio C, Read E: **The effects of authentic leadership, six areas of worklife, and occupational coping self-efficacy on new graduate nurses' burnout and mental health: A cross-sectional study**. *International journal of nursing studies* 2015, **52**:1080–1089.
61. Pisanti R, Lombardo C, Lucidi F, Lazzari D, Bertini M: **Development and validation of a brief Occupational Coping Self-Efficacy Questionnaire for Nurses**. *Journal of advanced nursing* 2008, **62**(2):238-247.
62. Martens ML: **A Comparison of Stress Factors in Home and Inpatient Hospice Nurses**. *Journal of hospice and palliative nursing* 2009, **11**(3):144-153.
63. Brunetto Y, Rodwell J, Shacklock K, Farr-Wharton R, Demir D: **The impact of individual and organizational resources on nurse outcomes and intent to quit**. *Journal of Advanced Nursing* 2016, **72**(12):3093-3103.
64. McHugh MD, Lake ET: **Understanding clinical expertise: nurse education, experience, and the hospital context**. *Research in nursing & health* 2010, **33**(4):276-287.

65. Schwarzer R, Jerusalem M: **Generalized Self-Efficacy scale**. In: *In J Weinman, S Wright, & M Johnston, Measures in health psychology: A user's portfolio Causal and control beliefs* Windsor, UK: NFER - Nelson; 1995: 35-37.
66. Hedeker D, Gibbons RD: **Longitudinal data analysis**, 1st edition edn: Wiley-Interscience; 2006.
67. Thompson C, Dalgleish L, Bucknall T, Estabrooks C, Hutchinson AM, Fraser K, Vos De R, Binnekade J, Barrett G, Saunders J: **The effects of time pressure and experience on nurses' risk assessment decisions: A signal detection analysis.** . *Nursing Research* 2008(57):302-311.
68. Nelson MF, Tarpey RJ: **Work scheduling satisfaction and work life balance of nurses: the perception of organizational justice**. *Academy of Health Care Management Journal* 2010, **6**(1):25-36.
69. Gillet N, Fouquereau E, Bonnaud-Antignac A, Mokounkolo R, Colombat P: **The mediating role of organizational justice in the relationship between transformational leadership and nurses' quality of work life: A cross-sectional questionnaire survey**. *International journal of nursing studies* 2013, **50**:1359–1367.
70. Baruch Y: **Response Rate in Academic Studies—A Comparative Analysis**. *Human Relations* 1999, **52**(4):421-438.

Figure 1 Click here to access/download;Figure;Fig 1 Relation modelled and perceived workload.tif

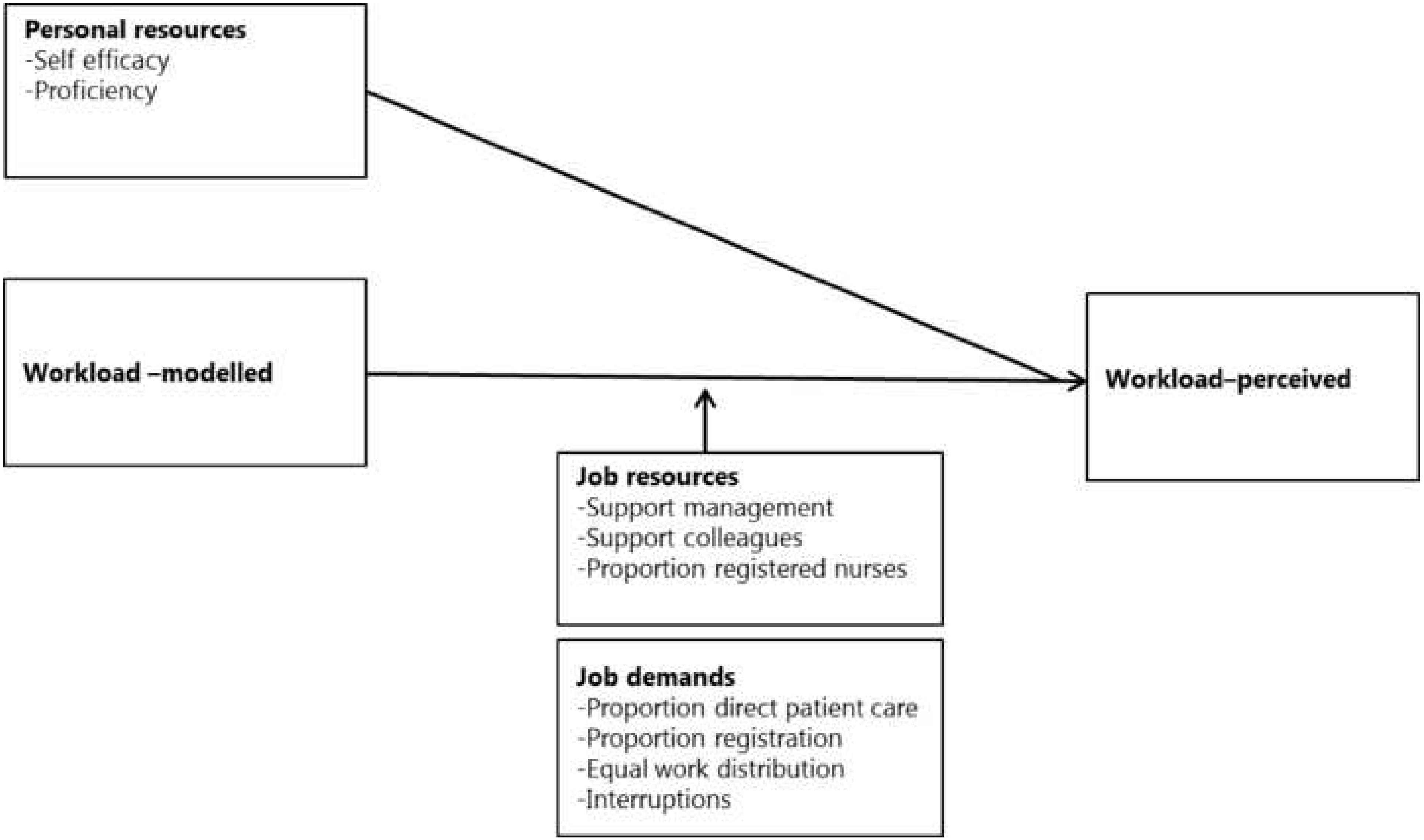

Figure 2

Click here to access/download;Figure;Fig 2 Average modelled workload per ward per day.tif

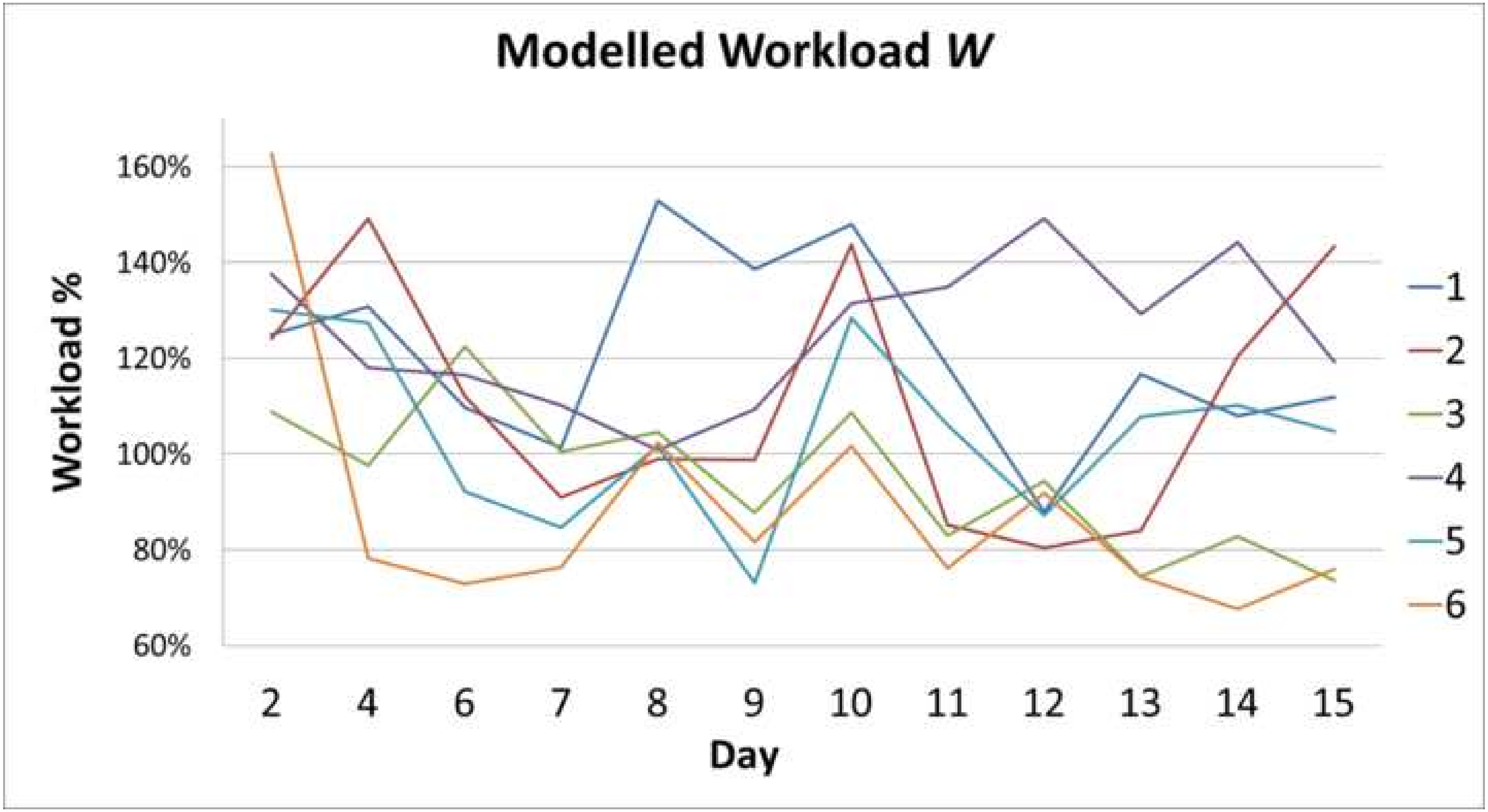

Figure 3

Click here to access/download;Figure;Fig 3 Five average perceived workloads.tif

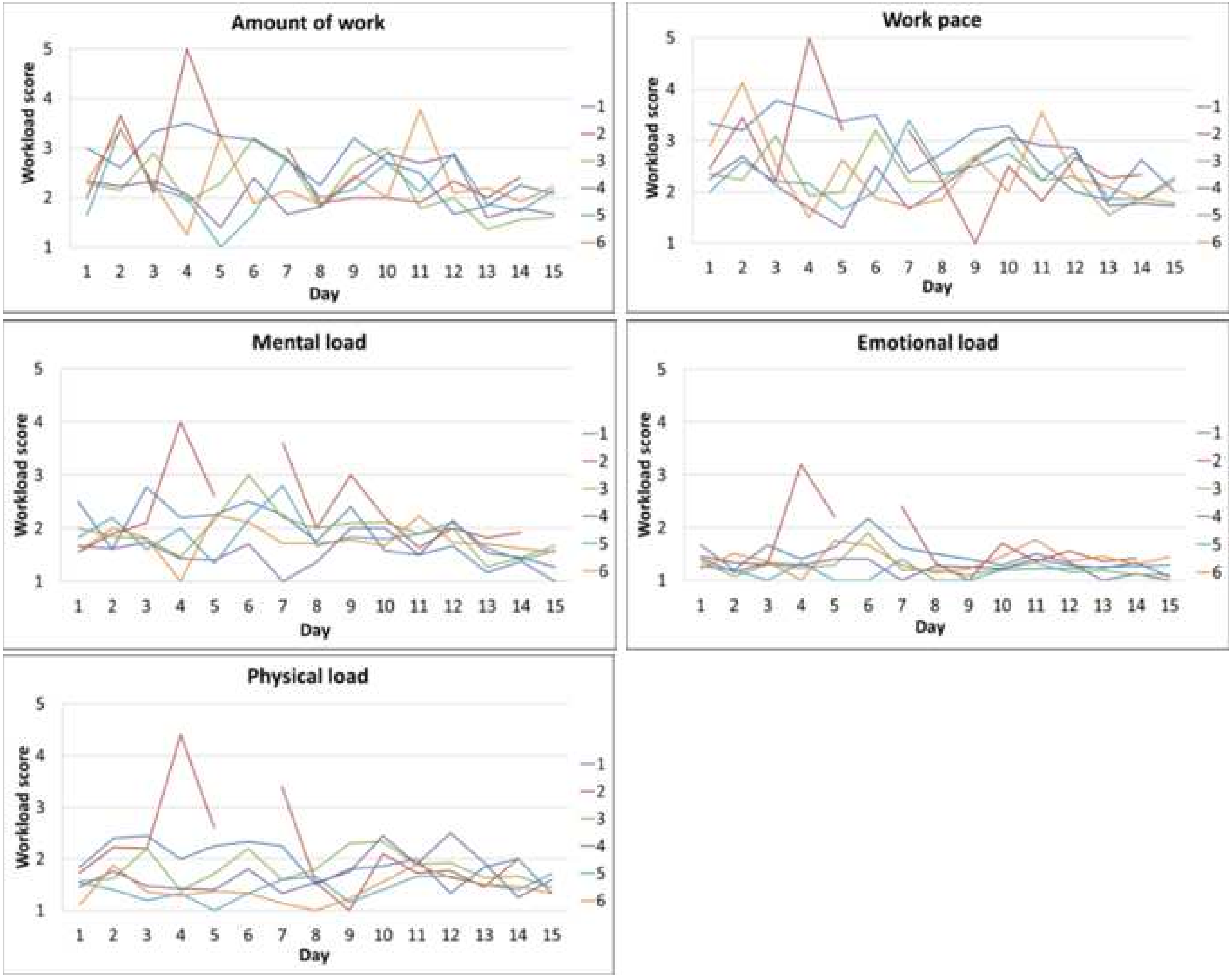

Click here to access/download
Supporting Information
Appendix 1_Mixed models experienced vs modelled workload.pdf

To:
Itamar Ashkenazi
Academic Editor
PLOS ONE

March 21st , 2020

Dear Dr. Itamar Ashkenazi,

Enclosed is a revision of a manuscript to be considered for publication in PLOS ONE. The manuscript was first submitted to you on December 14th 2019. A first review round has been returned to us on the 17th of February 2020. The review round resulted in a rejection of the manuscript in its current form, however, the option was given to re-submit the manuscript if the requested changes could be made. We have carefully looked at the reviewers' suggestions and would like to respond in this letter. We have addressed all points put forward by the referees and believe the manuscript has gained in clarity. Therefore, we would like to re-submit. Corrections have been made in the main manuscript, using 'Track changes' functionality. The new versions of the main document, with and without visible changes, have been re-submitted through your website. The following revisions have been made:

Reviewer 1:

1. *The perception of nurses workload compared to modeled workload is very important. The findings described no difference in workload and perceived workload. However, there are several limitations that prevent the findings from being generalizable. The most concerning is the inclusion of nursing students in the data set. Nursing students do not have active participation to the workforce.*
   In the Netherlands, nursing students do participate actively in the workforce, under supervision of registered nurses. Of course, they are not as proficient as fully qualified and experienced nurses but they do take on part of the workload and therefore need to be included in the model. In our study, we have determined proficiency levels for all types of nurses on the ward and have adjusted for variation in nurse proficiency in two ways:
   - The *modelled* workload was corrected for nurse proficiency (lines 316-322)
   - Effects of proficiency on *experienced* workload were also included in the study design (lines 234-240)
2. *Additionally, the manuscript refers to the use of nursing staff in registration, the term registration was not defined. What is meant by registration.*
   Registration is defined as all administrative and registration tasks done either on behalf of the patient (such as patient documentation, ordering medication and applying for tests and examinations) or as part of general administrative activities (such as ordering supplies, processing incoming mail). We have added the definition in the document, lines 372-375.
3. *Lastly, the manuscript describes numerous limitations which may have significant impact on study findings*. As stated in the limitations section, there are indeed limitations, however we have also shown that results of the study are significant, reliable and relevant to clinical practice.

Reviewer 2:

1. *In general, the manuscript is too dense which makes the reading really challenging. The approach and flow of the manuscript is confusing. There is too much information for a single manuscript, and it seems to me, a lot of work done behind these pages however, it is not properly presented. The manuscript should be reviewed for clarity. It will probably benefit from separation, i.e. a manuscript only to approach hypothesis 1 and 2.*
   We have rigorously adjusted the manuscript to improve clarity and flow of reading, especially throughout the introduction section.
   In our research, we study the relation between modelled and perceived workload, and the effects of several covariates on the relation. This is one coherent study, which means results need to be presented together, in one article.

4. *The Introduction section focuses too much on the authors previous work. Only the first paragraph mentions selected references on the issue of the study however, there is plenty of scientific literature on nursing workload, nursing intensity and complexity, that should be summarized and updated. I would suggest the authors to briefly introduce the concepts, summarize the evidence and present the rationale for the research question in a couple of pages. Their previous work might be presented apart as a*

supplementary appendix.

As suggested, the introduction has been updated with additional references and information on workload concepts. The introduction section has also been thoroughly rearranged to facilitate easy reading. Information on previous work of the study team has been condensed.

5. *The World Health Organization suggest to shift advancements of nursing approaches towards caring, moving from tasks to processes. It is a concern for this reviewer that a new workload measurement tool is not process-oriented but task-based. Does this make sense?* This is an understandable concern; the study team fully agrees and has no intention of reducing the complex nursing processes to separated tasks. When developing our workload management method, we put the nurses fully in the lead. The nurses indicated that a substantial part of their working day is determined by whether certain key characteristics apply to patients. Some patients require specific activities or attention that significantly influence the duration or complexity of the care processes and with that, required amount and type of nursing resources. The new workload management method includes the patient related tasks and processes that according to nurses would require considerable additional nursing time. Measures on complexity of care, time needed for non-patient related activities and required nurse proficiency are incorporated in the method. We have added concise information and references to previous research by our team that elaborates on the workload method.

6. *The Methods section is somehow confusion and incomplete. I would suggest to better specify the sub-sections: design, setting, participants (including selection criteria, sample and sampling), measures, data collection and data analysis.*
   We have rearranged and completed the Method section in line with the suggestions of the reviewer.

7. *Is the hospital in the setting of the study public or private?*
   Private, in the Netherlands most hospitals are. We have added this information to the setting, line 215

8. *In this same section, the authors wrote: "The study focused on the day shift workload, because this is the shift during which the most nursing activities are performed". Is this is a personal opinion?, It sounds like it was probably based on an historical bias, no longer trustworthy nowadays. Most university hospitals in Europe have afternoon surgery programs, so patients are readmitted to the wards late evening or early night. Most in-patients, either medical or surgical, are highly dependent during admission, among other reasons because of shortening length of stay. Their need for monitoring, surveillance, and assistance with their needs is probably the same during the day and the night shifts. Could the authors review the literature for any evidence supporting their statement?*
   De Cordova et al. (2014) found that "RN staffing was greater during the day than at night (4.3 HPPD vs. 3.4). However, the percentage of care hours provided by RNs at night was higher than the percentage of hours provided by RNs during the day (64.6% vs. 60.2%)."
   It is true that the percentage of direct patient care may be higher on other shifts. We noted that most nursing activities are done in the dayshift, because this is the shift that requires the most nursing staff. So understanding and balancing workload in this shift will have a big effect. In addition, there are nursing activities that are only done in the dayshift, for example standard visiting rounds and often also washing and mobilization of patients, wound care and discharge procedures. Also, when possible, complex nursing activities are planned in the dayshift, because there is more (senior) staff available to handle or supervise this, and medical staff is also easier to contact when necessary. So planned care is done as much as possible in the dayshift. This is the reason we chose to focus on the dayshift. We have added extra information on this in the setting section, line 217-218.

9. *No mention to the study ethical considerations, i.e informed consent. How were nurses invited to participate? Could they decline the invitation or it was something they "must" do.* The study was approved by the medical ethical review board of the hospital, as mentioned in line 400-403. Nurses that were not comfortable in being observed were allowed to work evening or night shifts, but almost none used this option: the study originated from the practical need of nurses to understand workload, so motivation to participate was high. Participation in filling out questionnaires was encouraged but on a voluntary basis. Response rates were high, as shown in Table 1.

10. *Lack of clarity at introducing the study aims and rigor at presenting the methods section prevents the findings to be considered.*
    The introduction and methods sections have been thoroughly reviewed and adjusted to address this issue

Reviewer 3:

1. *This manuscript discusses a very important issue. Its strength is that it provides both the evaluation of subjective perception along with the objective assessment of the workload on nursing work. This article is well structured methodologically and takes into account important variables that affect the subjective work concept. However, the authors need to acknowledge it is difficult to generalize the model to departments with unique nature, such as oncology, psychiatry, geriatrics, orthopedics, etc. This should be discussed in the limitations section.* We have added this limitation to the discussion section.

11. *The study period was 15 days. Although the authors noted that this was a relatively long study period, they should have considered measuring the workload during two distinct periods of time. A comparison of these two time periods could have strengthened their findings.*
Possibly, results would have been more powerful if the study was done over two separate time periods. However, due to practical issues we did not chose this option. If the study was set over two time periods, staff would need to be trained and informed twice (for example not all medical students would be available for both time periods and new nursing students would have entered the wards), equipment would need to be rented twice and the preparations such as barcoding patient rooms would need to be done again. This would have become too costly so the study team opted against this. This information has been added to the limitations section.

We thank you for all your help and suggestions so far, and look forward to your response.

Kind regards,
W.F.J.M. (Miranda) van den Oetelaar, MSc.

University Medical Center Utrecht
University of Utrecht
Heidelberglaan 100
3584CX Utrecht
The Netherlands
w.f.j.m.vandenoetelaar@umcutrecht.nl
Telephone +31 6 160 453 53
