# Supplementary material for: Exploring the relation between modelled and perceived workload of nurses and related job demands, job resources and personal resources; a longitudinal study
Source: PLoS One. 2021 Feb 26;16(2):e0246658. doi: 10.1371/journal.pone.0246658 (PMC7909623; doi:10.1371/journal.pone.0246658)
Supplement: Attachment — Submitted filename: Response to reviewers Manuscript PLOS ONE 20200319.docx [file pone.0246658.s001.docx]

**To:**

Itamar Ashkenazi

Academic Editor

PLOS ONE

March 21^st^ , 2020

Dear Dr. Itamar Ashkenazi,

Enclosed is a revision of a manuscript to be considered for publication in PLOS ONE. The manuscript was first submitted to you on December 14^th^ 2019. A first review round has been returned to us on the 17^th^ of February 2020. The review round resulted in a rejection of the manuscript in its current form, however, the option was given to re-submit the manuscript if the requested changes could be made. We have carefully looked at the reviewers’ suggestions and would like to respond in this letter. We have addressed all points put forward by the referees and believe the manuscript has gained in clarity. Therefore, we would like to re-submit. Corrections have been made in the main manuscript, using ‘Track changes’ functionality. The new versions of the main document, with and without visible changes, have been re-submitted through your website. The following revisions have been made:

**Reviewer 1:**

1. *The perception of nurses workload compared to modeled workload is very important. The findings described no difference in workload and perceived workload. However, there are several limitations that prevent the findings from being generalizable. The most concerning is the inclusion of nursing students in the data set. Nursing students do not have active participation to the workforce.*

In the Netherlands, nursing students do participate actively in the workforce, under supervision of registered nurses. Of course, they are not as proficient as fully qualified and experienced nurses but they do take on part of the workload and therefore need to be included in the model. In our study, we have determined proficiency levels for all types of nurses on the ward and have adjusted for variation in nurse proficiency in two ways:

- The *modelled* workload was corrected for nurse proficiency (lines 316-322)
- Effects of proficiency on *experienced* workload were also included in the study design (lines 234-240)

2. *Additionally, the manuscript refers to the use of nursing staff in registration, the term registration was not defined. What is meant by registration.*
   Registration is defined as all administrative and registration tasks done either on behalf of the patient (such as patient documentation, ordering medication and applying for tests and examinations) or as part of general administrative activities (such as ordering supplies, processing incoming mail). We have added the definition in the document, lines 372-375.
3. *Lastly, the manuscript describes numerous limitations which may have significant impact on study findings*. As stated in the limitations section, there are indeed limitations, however we have also shown that results of the study are significant, reliable and relevant to clinical practice.

**Reviewer 2**:

1. *In general, the manuscript is too dense which makes the reading really challenging. The approach and flow of the manuscript is confusing. There is too much information for a single manuscript, and it seems to me, a lot of work done behind these pages however, it is not properly presented. The manuscript should be reviewed for clarity. It will probably benefit from separation, i.e. a manuscript only to approach hypothesis 1 and 2.*
   We have rigorously adjusted the manuscript to improve clarity and flow of reading, especially throughout the introduction section.
   In our research, we study the relation between modelled and perceived workload, and the effects of several covariates on the relation. This is one coherent study, which means results need to be presented together, in one article.
4. *The Introduction section focuses too much on the authors previous work. Only the first paragraph mentions selected references on the issue of the study however, there is plenty of scientific literature on nursing workload, nursing intensity and complexity, that should be summarized and updated. I would suggest the authors to briefly introduce the concepts, summarize the evidence and present the rationale for the research question in a couple of pages. Their previous work might be presented apart as a supplementary appendix.*
   As suggested, the introduction has been updated with additional references and information on workload concepts. The introduction section has also been thoroughly rearranged to facilitate easy reading. Information on previous work of the study team has been condensed.
5. *The World Health Organization suggest to shift advancements of nursing approaches towards caring, moving from tasks to processes. It is a concern for this reviewer that a new workload measurement tool is not process-oriented but task-based. Does this make sense?* This is an understandable concern; the study team fully agrees and has no intention of reducing the complex nursing processes to separated tasks. When developing our workload management method, we put the nurses fully in the lead. The nurses indicated that a substantial part of their working day is determined by whether certain key characteristics apply to patients. Some patients require specific activities or attention that significantly influence the duration or complexity of the care processes and with that, required amount and type of nursing resources. The new workload management method includes the patient related tasks and processes that according to nurses would require considerable additional nursing time. Measures on complexity of care, time needed for non-patient related activities and required nurse proficiency are incorporated in the method. We have added concise information and references to previous research by our team that elaborates on the workload method.
6. *The Methods section is somehow confusion and incomplete. I would suggest to better specify the sub-sections: design, setting, participants (including selection criteria, sample and sampling), measures, data collection and data analysis.*

We have rearranged and completed the Method section in line with the suggestions of the reviewer.

7. *Is the hospital in the setting of the study public or private?*
   Private, in the Netherlands most hospitals are. We have added this information to the setting, line 215
8. *In this same section, the authors wrote: “The study focused on the day shift workload, because this is the shift during which the most nursing activities are performed”. Is this is a personal opinion?, It sounds like it was probably based on an historical bias, no longer trustworthy nowadays. Most university hospitals in Europe have afternoon surgery programs, so patients are readmitted to the wards late evening or early night. Most in-patients, either medical or surgical, are highly dependent during admission, among other reasons because of shortening length of stay. Their need for monitoring, surveillance, and assistance with their needs is probably the same during the day and the night shifts. Could the authors review the literature for any evidence supporting their statement?*

*De Cordova et al. (2014) found that “RN staffing was greater during the day than at night (4.3 HPPD vs. 3.4). However, the percentage of care hours provided by RNs at night was higher than the percentage of hours provided by RNs during the day (64.6% vs. 60.2%).”*
It is true that the percentage of direct patient care may be higher on other shifts. We noted that most nursing activities are done in the dayshift, because this is the shift that requires the most nursing staff. So understanding and balancing workload in this shift will have a big effect. In addition, there are nursing activities that are only done in the dayshift, for example standard visiting rounds and often also washing and mobilization of patients, wound care and discharge procedures. Also, when possible, complex nursing activities are planned in the dayshift, because there is more (senior) staff available to handle or supervise this, and medical staff is also easier to contact when necessary. So planned care is done as much as possible in the dayshift. This is the reason we chose to focus on the dayshift. We have added extra information on this in the setting section, line 217-218.

9. *No mention to the study ethical considerations, i.e informed consent. How were nurses invited to participate? Could they decline the invitation or it was something they “must” do.* The study was approved by the medical ethical review board of the hospital, as mentioned in line 400-403. Nurses that were not comfortable in being observed were allowed to work evening or night shifts, but almost none used this option: the study originated from the practical need of nurses to understand workload, so motivation to participate was high. Participation in filling out questionnaires was encouraged but on a voluntary basis. Response rates were high, as shown in Table 1.
10. *Lack of clarity at introducing the study aims and rigor at presenting the methods section prevents the findings to be considered.*
    The introduction and methods sections have been thoroughly reviewed and adjusted to address this issue

**Reviewer 3**:

1. *This manuscript discusses a very important issue. Its strength is that it provides both the evaluation of subjective perception along with the objective assessment of the workload on nursing work. This article is well structured methodologically and takes into account important variables that affect the subjective work concept. However, the authors need to acknowledge it is difficult to generalize the model to departments with unique nature, such as oncology, psychiatry, geriatrics, orthopedics, etc. This should be discussed in the limitations section.* We have added this limitation to the discussion section.
11. *The study period was 15 days. Although the authors noted that this was a relatively long study period, they should have considered measuring the workload during two distinct periods of time. A comparison of these two time periods could have strengthened their findings.*

Possibly, results would have been more powerful if the study was done over two separate time periods. However, due to practical issues we did not chose this option. If the study was set over two time periods, staff would need to be trained and informed twice (for example not all medical students would be available for both time periods and new nursing students would have entered the wards), equipment would need to be rented twice and the preparations such as barcoding patient rooms would need to be done again. This would have become too costly so the study team opted against this. This information has been added to the limitations section.

We thank you for all your help and suggestions so far, and look forward to your response.

Kind regards,

W.F.J.M. (Miranda) van den Oetelaar, MSc.

University Medical Center Utrecht

University of Utrecht

Heidelberglaan 100

3584CX Utrecht

The Netherlands

w.f.j.m.vandenoetelaar@umcutrecht.nl

Telephone +31 6 160 453 53
